# Cooperation between bHLH transcription factors and histones for DNA access

**DOI:** 10.1038/s41586-023-06282-3

**Published:** 2023-07-05

**Authors:** Alicia K. Michael, Lisa Stoos, Priya Crosby, Nikolas Eggers, Xinyu Y. Nie, Kristina Makasheva, Martina Minnich, Kelly L. Healy, Joscha Weiss, Georg Kempf, Simone Cavadini, Lukas Kater, Jan Seebacher, Luca Vecchia, Deyasini Chakraborty, Luke Isbel, Ralph S. Grand, Florian Andersch, Jennifer L. Fribourgh, Dirk Schübeler, Johannes Zuber, Andrew C. Liu, Peter B. Becker, Beat Fierz, Carrie L. Partch, Jerome S. Menet, Nicolas H. Thomä

**Affiliations:** 1 Friedrich Miescher Institute for Biomedical Research, Basel, Switzerland; 2 University of Basel, Basel, Switzerland; 3 Department of Chemistry and Biochemistry, University of California, Santa Cruz, Santa Cruz, CA USA; 4 Biomedical Center, Molecular Biology Division, Ludwig-Maximilians-Universität, Munich, Germany; 5 Department of Biology, Center for Biological Clock Research, Texas A&M University, College Station, TX USA; 6 Institute of Chemical Sciences and Engineering, Ecole Polytechnique Fédérale de Lausanne, Lausanne, Switzerland; 7 Research Institute of Molecular Pathology, Vienna BioCenter, Vienna, Austria; 8 Department of Physiology and Aging, College of Medicine, University of Florida, Gainesville, FL USA; 9 Medical University of Vienna, Vienna, Austria; 10 Present Address: University of Basel, Basel, Switzerland

**Keywords:** Cryoelectron microscopy, Nucleosomes, Transcriptional regulatory elements

## Abstract

The basic helix–loop–helix (bHLH) family of transcription factors recognizes DNA motifs known as E-boxes (CANNTG) and includes 108 members^1^. Here we investigate how chromatinized E-boxes are engaged by two structurally diverse bHLH proteins: the proto-oncogene MYC-MAX and the circadian transcription factor CLOCK-BMAL1 (refs. ^2,3^). Both transcription factors bind to E-boxes preferentially near the nucleosomal entry–exit sites. Structural studies with engineered or native nucleosome sequences show that MYC-MAX or CLOCK-BMAL1 triggers the release of DNA from histones to gain access. Atop the H2A–H2B acidic patch^4^, the CLOCK-BMAL1 Per-Arnt-Sim (PAS) dimerization domains engage the histone octamer disc. Binding of tandem E-boxes^5–7^ at endogenous DNA sequences occurs through direct interactions between two CLOCK-BMAL1 protomers and histones and is important for circadian cycling. At internal E-boxes, the MYC-MAX leucine zipper can also interact with histones H2B and H3, and its binding is indirectly enhanced by OCT4 elsewhere on the nucleosome. The nucleosomal E-box position and the type of bHLH dimerization domain jointly determine the histone contact, the affinity and the degree of competition and cooperativity with other nucleosome-bound factors.

## Abstract (web summary)

Cryo-EM structures and analysis provide insight into the mechanisms by which basic helix–loop–helix transcription factors access E-box DNA sequences that are embedded within nucleosomes, and cooperate with other transcription factors.

## Main

The human bHLH transcription factor (TF) family consists of 108 members that form pairs of homo- and heterodimers^1,8^. Members of the bHLH family control essential biological processes ranging from cell growth, proliferation and metabolism^9^, neurogenesis^10^ and myogenesis^11^, to the response to hypoxia^12^, and circadian rhythms^13,14^. The bHLH DNA-binding fold contains an N-terminal basic helix that interacts with the major groove of DNA, followed by a loop and a second α-helix^15^. bHLH DNA-binding domains can be adjoined to different types of dimerization domains such as leucine zipper (LZ) domains (for example, MYC, MAX and MAD), PAS domains (for example, CLOCK, BMAL1 and HIF1α) or orange domains (for example, HES1–HES7)^1^. Different families of bHLH proteins recognize a core DNA motif called the Ephrussi or Enhancer-box (E-box), which is a short palindromic sequence with a degenerate CANNTG motif, present around 15 million times in the human genome^16^. We focused on two structurally and evolutionarily distinct bHLH members from the bHLH-LZ and bHLH-PAS clades, represented by the proliferation regulator MYC-MAX and the circadian TF CLOCK-BMAL1, respectively.

The proto-oncogene MYC has an essential role in the cell’s circuitry to regulate cell growth^17^. Most tumour types show deregulated expression of *MYC* owing to direct alterations of the locus (for example, gene amplification or translocation) or from the activation of upstream signalling pathways (Wnt, Notch and so on), resulting in MYC-driven oncogenic transformation^18^. As a transcriptional activator, MYC works with MAX (hereafter MYC-MAX). MAX, in turn, forms homodimers and heterodimers with other bHLH-LZ proteins MXD1–MXD4, MNT and MGA that function as transcriptional repressors^9^.

The heterodimeric bHLH-PAS TF CLOCK-BMAL1 is a crucial component of the molecular clock that confers an approximately 24-hour period for rhythmic expression of nearly 40% of the genome (across tissues), including essential genes in metabolism, hormone secretion and the cell cycle^19,20^. CLOCK-BMAL1 interacts with E-box elements and coregulators, including the dedicated circadian repressors Period (PER) and Cryptochrome (CRY), to drive transcriptional oscillations throughout the day^21^.

An essential regulatory mechanism that governs the access of TFs to genomic target sites is the chromatin environment, in which nucleosomes restrict TF binding to DNA^22,23^. It is estimated that bHLH proteins bind less than 1% of total E-boxes at a given time^24^. However, the mechanisms by which single bHLH TFs read out nucleosome-embedded E-boxes within chromatin, and by which bHLH members cooperate with other TFs, are unknown.

We set out to address how different classes of bHLH TFs, MYC-MAX and CLOCK-BMAL1, together with an unrelated TF, OCT4, structurally and functionally interact with nucleosomes.

## Histones impose restrictions on DNA access

**Fig. 1:**
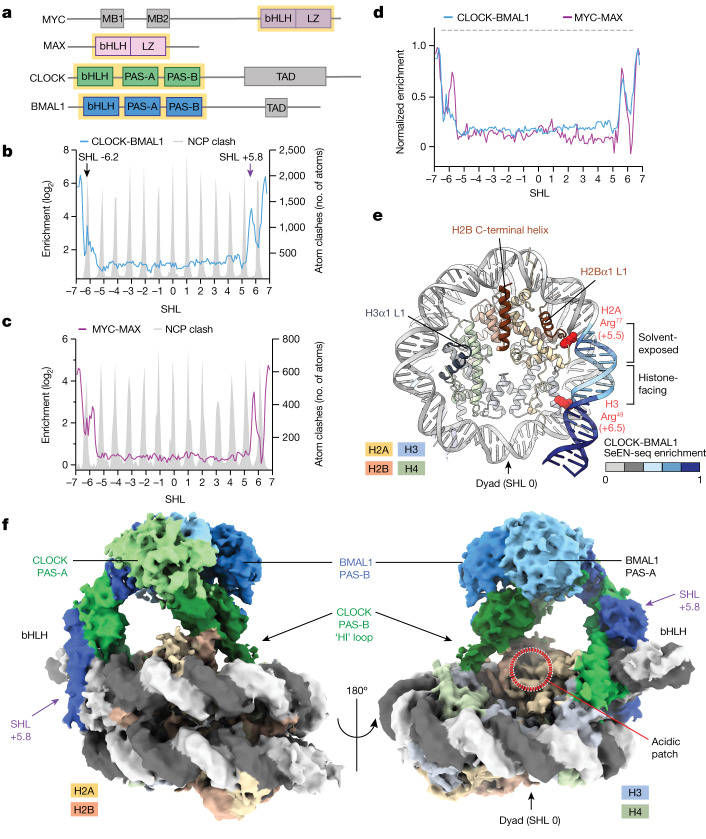
CLOCK-BMAL1 and MYC-MAX are nucleosome end-binders. **a**, Domain schematic of bHLH TFs. The yellow box highlights the construct boundaries used in this study. MB1, MYC box 1; MB2, MYC box 2; TAD, transactivation domain. **b**,**c**, SeEN-seq profile of CLOCK-BMAL1 (mouse CLOCK residues 26–395; mouse BMAL1 residues 62–441) (**b**) or MYC-MAX (human MYC residues 351–437; human MAX residues 22–102) (**c**). The predicted atomic clash of the corresponding TF with the NCP is overlaid (grey). Values are shown as an average of independent replicates (*n* = 3). The SHLs indicate where the DNA major groove faces towards the histones. The indicated SHL of the E-box corresponds to the centroid of the motif CACGTG (see also Methods). Internal sites are defined as positions with a free energy of DNA unwrapping greater than around 1.2 kcal mol^−1^ between SHL−5 and SHL+5 (refs. ^54,55^). **d**, Overlay of CLOCK-BMAL1 SeEN-seq profile with MYC-MAX. The highest value of each enrichment profile is normalized to 1. Dashed grey lines indicate regions of high atomic clash for both TFs. **e**, Structure of a human NCP (Protein Data Bank (PDB): 6T93) with the DNA coloured according to the normalized CLOCK-BMAL1 SeEN-seq profile. ‘Hotspots’ of histone interaction are annotated^4,28^. **f**, Cryo-EM map of CLOCK-BMAL1 bound to an E-box motif at SHL+5.8.

**Extended Data Fig. 1:**
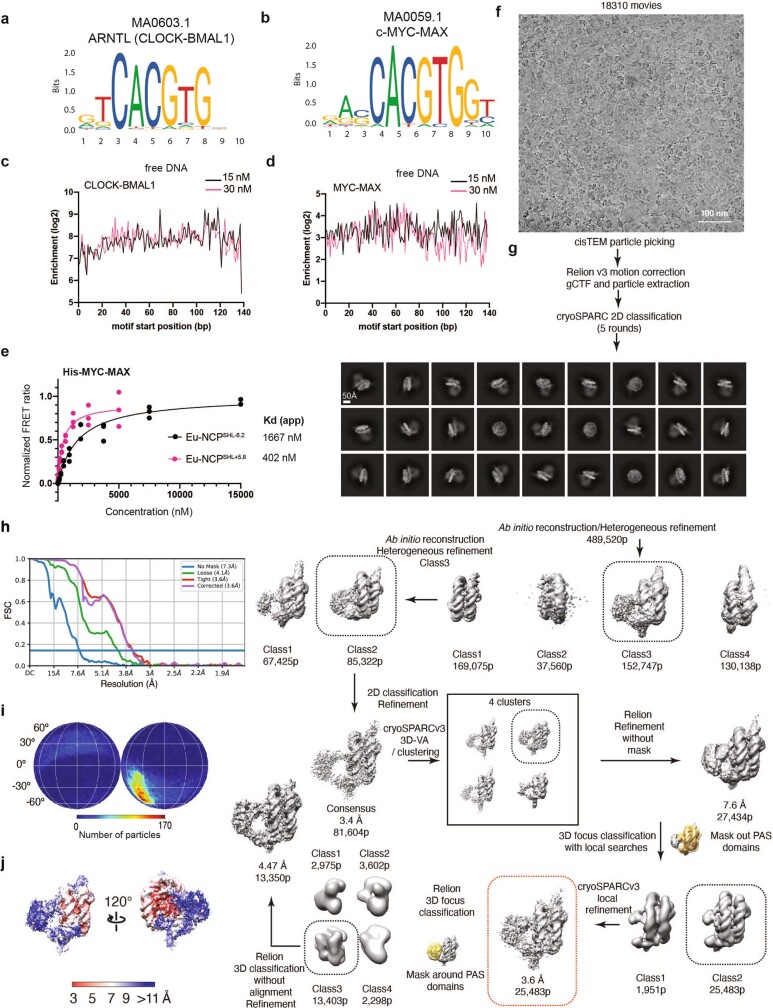
Interactions between bHLH TFs and E-boxes are influenced by histones. **a**, Motif logo for BMAL1 (ARNTL) from the Jaspar database^27^. **b**, MYC motif logo from the Jaspar database. **c**,**d**, SeEN-seq enrichment profile of CLOCK-BMAL1 bHLH PAS-AB (**c**) and MYC-MAX (**d**) in the presence of the free DNA library pool (no histones) at two different protein concentrations, 15 and 30 nanomolar (nM). The same DNA library was used to assemble nucleosomes and perform SeEN-seq as in Fig. 1b, c. **e**, Binding preferences in TR-FRET are consistent with enrichment in SeEN-seq, where MYC-MAX shows a higher enrichment at SHL+5.8 (log2: 3.5) versus SHL-6.2 (log2: 2.2). Incubation of biotinylated NCPs (NCP^SHL-6.2^ and NCP^SHL+5.8^) with LANCE Eu-W8044 streptavidin (donor) with increasing amounts of His-MYC-MAX bound by an Ultra ULight α-6×His antibody (acceptor). Three technical replicates are shown for each condition and three biological replicates were performed with similar results. The signal was corrected for direct acceptor excitation by subtracting the signal observed in the absence of the nucleosome. The resulting raw signals were fitted to the Bmax values of 1 using a one-site specific binding model using Prism 7 (GraphPad). **f**, Representative cryo-EM micrograph of 18,310 individual micrographs collected. Denoised with Janni^81^. **g**, See Methods. The movies were pre-processed within cryoFLARE and the resulting micrographs were imported in cisTEM for particle picking. 3D variability analysis (cryoSPARC v.3) in combination with 3D classification (RELION) resulted in a homogeneous subset of particles that were used for the final 3D reconstruction. The boxes defined by a dashed line indicate the good models and set of particles used for the following step in the data processing workflow. **h**, Gold-standard FSC curve for the 3.6 Å resolution map is highlighted by the red dashed box in **g**. **i**, Angular distribution for the particles leading to the 3.6 Å resolution map. **j**, Local-resolution filtered map (MonoRes) for the 3.6 Å resolution map highlighted by the red dashed box shown in **g** (ref. ^90^).

We first examined how bHLH TFs access nucleosome-embedded E-boxes using SeEN-seq^25^: a single E-box core motif (GGCACGTGTC) bound both by CLOCK-BMAL1 and MYC-MAX^26^ (Extended Data Fig. 1a,b) is tiled at one-base-pair (bp) intervals throughout all registers of a nucleosome pool (E-box nucleosome core particle (NCP)) using a Widom 601 sequence (W601) variant^26,27^ devoid of E-box motifs (Supplementary Table 1). CLOCK-BMAL1 and MYC-MAX were incubated at varying concentrations with the E-box NCP pool (Fig. 1a). The slow-migrating TF–nucleosome complexes (bound) and fast-migrating nucleosomes (unbound) were separated by native PAGE electrophoresis and extracted. Comparison of the next-generation sequencing (NGS) reads of the bound and unbound species resulted in a relative enrichment profile for each motif position throughout the nucleosome (Extended Data Fig. 1c,d). The MYC-MAX and CLOCK-BMAL1 SeEN-seq profiles show end-binding behaviour, preferentially at E-box sites at superhelical locations (SHLs)+/−7 to SHLs+/−5 (Fig. 1b–e). Binding was attenuated at more internal sites, between SHL−5 and SHL+5. The high accessibility regions at SHL+5.5 to SHL+7 are shared between MYC-MAX and CLOCK-BMAL1, whereas peaks at SHL−6.5 to SHL−5.5 differed in position and relative affinity (Extended Data Fig. 1e). Accessibility peaks for MYC-MAX and CLOCK-BMAL1 generally coincide with solvent-facing E-box positions, where fewer steric clashes are expected (Fig. 1b,c).

## CLOCK-BMAL1 displaces nucleosomal DNA

**Fig. 2:**
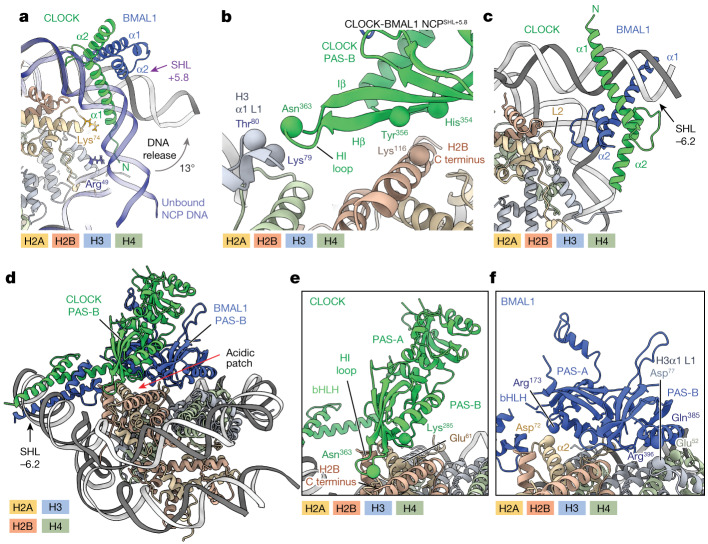
The PAS domains of CLOCK-BMAL1 interface with the histones. **a**, The CLOCK-BMAL1 bHLH domain bound to SHL+5.8 releases DNA. **b**, Magnified view of the PAS-B domain of CLOCK at the histone interface (see also Fig. 1f). **c**, CLOCK-BMAL1 bHLH domain bound at SHL−6.2. The CLOCK-BMAL1 PAS domains are removed for clarity. **d**, Atomic model of CLOCK-BMAL1 bound to a nucleosomal E-box at SHL−6.2. **e**, Magnified view of CLOCK bound at SHL−6.2. The BMAL1 chain is removed for clarity. **f**, Magnified view of BMAL1 at SHL−6.2. The CLOCK chain is removed.

**Extended Data Fig. 2:**
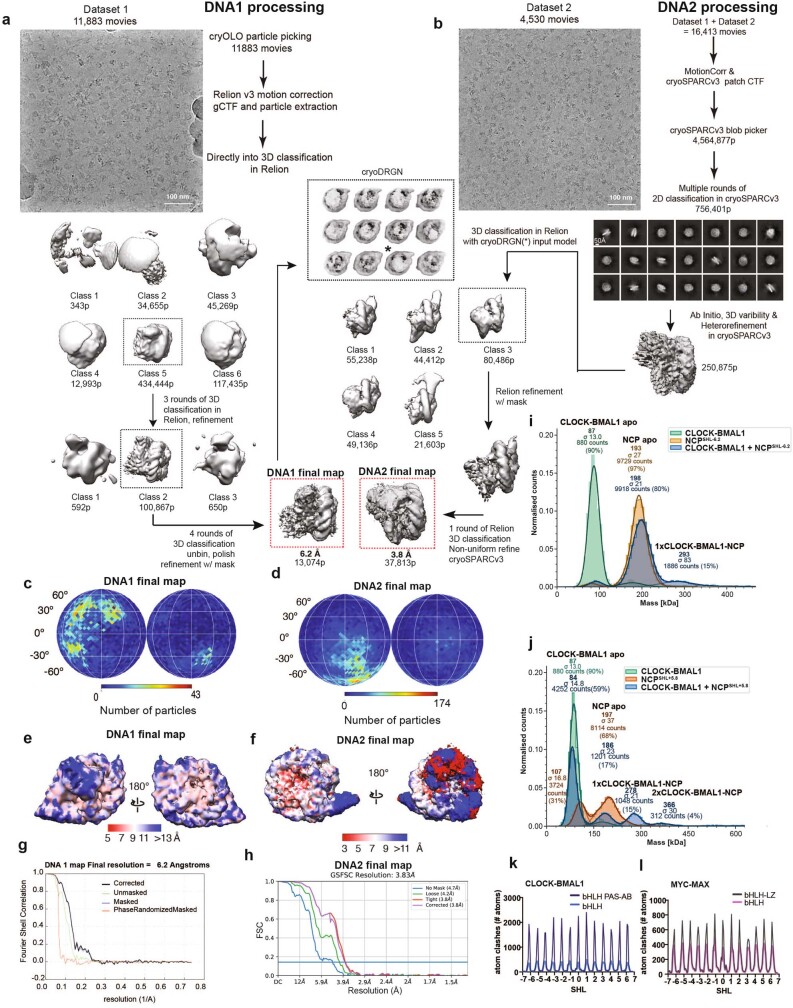
CLOCK-BMAL1-NCP^SHL-6.2^ cryo-EM processing. **a**,**b**, Representative cryo-EM micrographs for datasets 1 (**a**) and 2 (**b**) denoised with Janni and processing (see also Methods). The movies of dataset 1 were pre-processed within cryoFLARE and the particles were picked using crYOLO^81^. Multiple rounds of 3D classification (RELION 3.1). The boxes defined by a dashed line indicate the good models and set of particles used for the following step in the workflow. Particles from a classification in RELION (100,867 particles) were further analysed using cryoDRGN^110^, and the map indicated in the asterisk (*) was used as an input model for 3D classification of the combined datasets 1 and 2. After merging, particles were picked with cryoSPARC v.3 blob picker. Multiple rounds of 2D classification (cryoSPARC v.3) and 3D classification yielded a homogeneous subset of particles. **c**,**d**, Angular distribution for the particles leading to the 6.2 Å (**c**) and 3.8 Å (**d**) resolution map. **e**,**f**, Local-resolution filtered map (MonoRes) for the 6.2 Å (**e**) and 3.8 Å (**f**) resolution map. **g**,**h**, Gold-standard FSC curve for the 6.2 Å (**g**) and 3.8 Å (**h**) resolution map. **i**,**j** Molecular mass distribution histogram of CLOCK-BMAL1-NCP^SHL-6.2^ (**i**) and CLOCK-BMAL1-NCP^SHL+5.8^ (**j**). CLOCK-BMAL1 and the nucleosomes were first measured individually at 20 nM and in a 1:6 ratio. CLOCK-BMAL1 and NCP^SHL-6.2^ form a 1:1 complex, whereas for NCP^SHL+5.8^ a minority species with a 1:2 stoichiometry is also observed. **k**, The CLOCK-BMAL1 bHLH domain only free-DNA-bound structure (PDB: 4h10) or the composite bHLH-PAS-AB model (PDB: 4F3L, 4H10) was superimposed on a nucleosome template model (PDB: 6T93) in all DNA registers, and a clash score was calculated as the total number of atoms in the bHLH domain closer than 1 Å to nucleosome atoms (see also Methods). **l**, The clash score of the MYC-MAX bHLH domain only (PDB: 1NKP, Uniprot human residues 351–411 for MYC, 22–54 for MAX) or the composite bHLH-LZ model (PDB: 1NKP, entire chains of one heterodimer) to the nucleosome was calculated as in **k**.

To dissect the molecular basis of CLOCK-BMAL1 binding throughout the nucleosome, we determined cryo-electron microscopy (cryo-EM) structures of CLOCK-BMAL1 bound to a solvent-exposed motif at SHL+5.8 (CLOCK-BMAL1-NCP^SHL+5.8^) with an overall resolution of 3.6 Å (Figs. 1f and 2a,b and Extended Data Fig. 1f–j), and at a histone-facing E-box at SHL−6.2 (CLOCK-BMAL1-NCP^SHL−6.2^) at 3.8 Å (Fig. 2c–f, Extended Data Fig. 2a–j and Extended Data Table 1). The resolution around the NCP was 3–5 Å, whereas the CLOCK and BMAL1 PAS domains were between 9 Å and 11 Å, with sufficient features to confidently place all domains.

**Extended Data Fig. 3:**
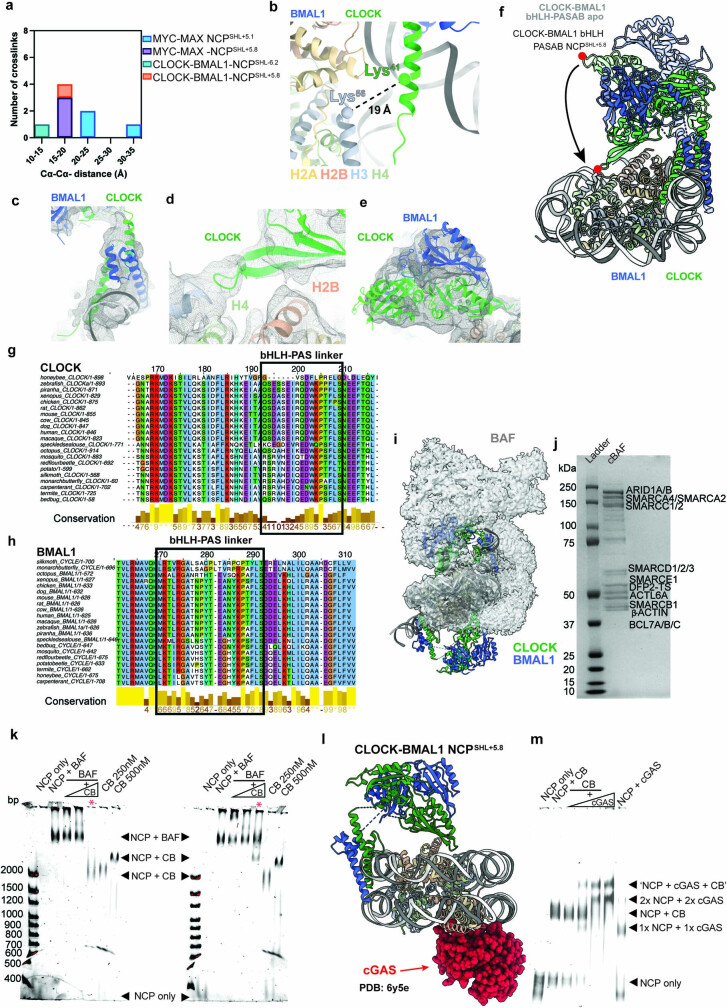
CLOCK-BMAL1 competes with chromatin binders that bind both acidic patches. **a**, Bar graph showing the number of cross-links obtained for the cryo-EM structures as a function of the obtained cross-link distances. **b**, Cross-link between histone H3 and CLOCK bHLH lysines (spheres). The cross-linker was DSSO and indicated distances (dashes) are between lysine Cα atoms. **c**–**e**, Map density around CLOCK-BMAL1 bHLH (**c**), interface between CLOCK PAS-B HI loop and H3α1 L1 (**d**) and PAS domains (**e**) at position SHL+5.8. The contour levels are 5.98 (**c**), 5.92 (**d**) and 5.86 (**e**). Maps were postprocessed by low-pass filtering or model-based local amplitude scaling (LocScale)^88^. **f**, Alignment of the CLOCK-BMAL1 bHLH-PAS-AB crystal structure (apo) onto the CLOCK-BMAL1 bHLH-PAS-AB-nucleosome-bound structure at SHL+5.8. The alignment was performed by Needleman-Wunsch using the bHLH residues 29–89 of CLOCK in ChimeraX. The interaction of the PAS domains with the histone octamer is accommodated by flexible linkers (22 residues in BMAL1, 17 residues in CLOCK) connecting the PAS-AB domains and the bHLH domains. **g**,**h**, Sequence alignment of CLOCK (**g**) and BMAL1 (**h**) proteins across species using a multiple sequence alignment^111^. Amino acid conservation is coloured according to Clustal using JalView^112^. **i**, Overlay of CLOCK-BMAL1 at SHL±5.8 with the map of a BAF-bound nucleosome (EMD-0974). **j**, SDS–PAGE of BAF after size-exclusion chromatography. **k**, EMSA competition assays between CLOCK-BMAL1 (CB) and BAF. The NCP (20 nM) was incubated with either, BAF only (100 nM), BAF (100 nM) with increasing amounts of CLOCK-BMAL1 (125 nM, 250 nM and 500 nM) or with CLOCK-BMAL1 only (250 nM, 500 nM). Three independent replicates were performed and two representative EMSAs are shown. Asterisk (*) indicates the lane where competition is most evident with the appearance of a CLOCK-BMAL1-NCP complex. **l**, Model of CLOCK-BMAL1 (at SHL+5.8) and cGAS (PDB: 6y5e) co-binding a nucleosome. **m**, EMSA competition assays between CLOCK-BMAL1 and the immune signalling sensor cGAs. The NCP was incubated with either CLOCK-BMAL1 (250 nM), CLOCK-BMAL1 with increasing amounts of cGAS (18.75 nM, 37.5 nM, 75 nM and 150 nM) or cGAS (75 nM). 3 independent biological replicates were performed, and one representative replicate is shown. A higher-running band that is likely to correspond to a higher-order CLOCK-BMAL1-cGAS-NCP complex is observed when titrating cGAS to the CLOCK-BMAL1-NCP complex.

In the CLOCK-BMAL1-NCP^SHL+5.8^ structure, the nucleosomal DNA is distorted to accommodate CLOCK-BMAL1, consistent with the E-box not being fully accessible to the bHLH DNA-binding fold (Fig. 1f and Extended Data Fig. 2k,l). The CLOCK-BMAL1 bHLH fold is oriented perpendicular to the plane of the nucleosomal disc. It binds the solvent-facing E-box by separating the DNA from histones H3 and H2A over around 17 bp from SHL+7.5 to SHL+5.5 (Figs. 1f and 2a). Residues H3 Arg^49^ and H2A Lys^74^, which engage the nucleosomal DNA duplex in the uncomplexed nucleosome structure, are orphaned in the presence of CLOCK-BMAL1 (ref. ^28^) (Fig. 2a). Cross-linking mass spectrometry (XL-MS) confirmed the assignment with the N-terminal basic helix of the CLOCK bHLH domain sandwiched between histone H2A loop 2 (L2) and the DNA duplex (Extended Data Fig. 3a–c and Supplementary Table 2).

In addition to the bHLH–H2A interaction, we observe a more prominent TF–histone interface (around 300 Å^2^) between the PAS domains of CLOCK and histones H2B, H3 and H4, made possible by the flexible linkers between the PAS-AB and bHLH domains (Fig. 2b and Extended Data Fig. 3d–h). The CLOCK PAS domains bind to the H2B C-terminal helix and the junction between the H3 α1 helix and its L1 loop (designated H3α1 L1 elbow)^4^.

CRY1 and CRY2 exert their potent activity through direct interactions with the CLOCK HI loop (residues 361–364) connecting the Hβ and Iβ strands—an interaction that is crucial for completing the daily transcription–translation feedback loop^29,30^. The CLOCK PAS-B HI loop is adjacent to the H3α1 L1 elbow (Lys^79^ and Thr^80^) and is immersed in interactions with the histone core, implying that histone engagement by CLOCK-BMAL1 spatially competes with CRY binding (Fig. 2b).

Proteins that bind nucleosomes through protein–protein interactions frequently engage one of two acidic patches comprised of histones H2A (Glu^61^, Asp^90^ and Glu^92^) and H2B (Glu^105^ and His^109^)^4,31^. The CLOCK-BMAL1 PAS footprint blocks one acidic patch, leading to expected clashes with the chromatin remodeller BRG/BRM-associated factor (BAF) complex, which engages both patches^32,33^ (Fig. 1f and Extended Data Fig. 3i). Accordingly, BAF and CLOCK-BMAL1 compete in electrophoretic mobility shift assay (EMSA) experiments for nucleosome binding (Extended Data Fig. 3j,k). By contrast, the innate immunity sensor cGAS occupies only one acidic patch^34^ and exhibits EMSA shift patterns consistent with co-occupying nucleosomes with CLOCK-BMAL1 (Extended Data Fig. 3l,m). CLOCK-BMAL1 binding at SHL+5.8 is therefore incompatible with chromatin binders that engulf nucleosomes but compatible with single acidic patch binders that bind nucleosomes along with CLOCK-BMAL1.

## The E-box register specifies interactions

**Extended Data Fig. 4:**
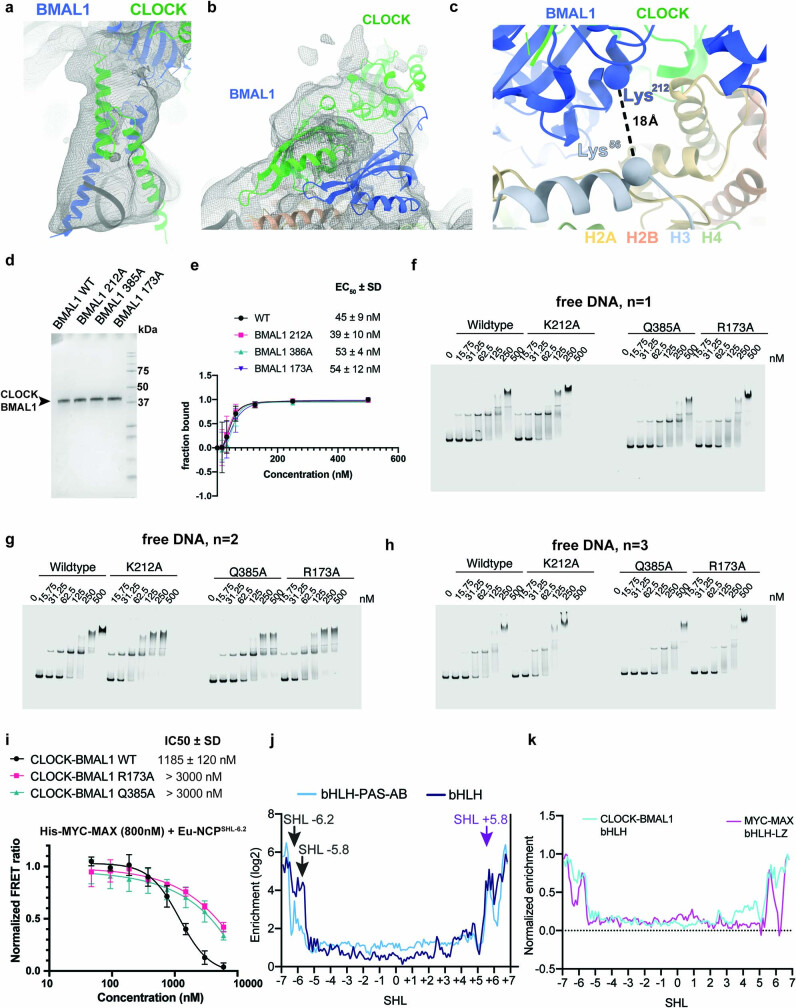
The PAS domains of CLOCK-BMAL1 contribute specifically to nucleosome binding. **a**,**b**, Map density around CLOCK-BMAL1 (**a**) bHLH and (**b**) PAS domains. The contour levels are 0.00192 (**a**) and 0.00137 (**b**). Maps were postprocessed by low-pass filtering or model-based local amplitude scaling (LocScale)^88^. **c**, Cross-link between BMAL1 PAS-A and histone H3 lysines (spheres). The cross-linker was DSSO and distances (dashes) are between lysine Cα atoms. **d**, The CLOCK-BMAL1 bHLH PAS-AB heterodimer wild-type (WT) and mutants (K212A, Q385A, R173A) were purified (Methods) and equal concentrations (1 µM, 10 µl) were analysed by SDS–PAGE and stained with Coomassie. Subsequent EMSAs and FRET were performed assuming these concentrations. **e**, BMAL1 mutations K212A, Q385A and R173A have minimal effect on free DNA binding. Quantification of free DNA binding (*n* = 3 biological replicates shown as mean ±SD) to the Cy5-labelled-SHL-6.2 DNA sequence using electrophoretic mobility shift assays (EMSA) in the presence of CLOCK-BMAL1 bHLH-PAS-AB WT or mutant proteins as seen in **d**. The three biological replicates are shown in **f**–**h**. Gels were imaged using a Licor instrument and quantified using the Empiria software package. The fraction bound is calculated as a percentage of the unbound probe. **i**, BMAL1 mutations Q385A and R173A show reduced nucleosome binding as compared to wild-type. TR-FRET counter-titration of unlabelled CLOCK-BMAL1 WT and mutants into the preassembled Eu-NCP^SHL-6.2^-His-MYC-MAX complex. Three technical replicates are shown for each condition, and three biological replicates were performed with similar results. **j**, SeEN-seq of CLOCK-BMAL1 containing the PAS domains (bHLH PAS-AB) and the bHLH region only (bHLH). **k**, Overlay of the CLOCK-BMAL1 bHLH only SeEN-seq with MYC-MAX bHLH LZ (as shown in Fig. 1d). The highest value of each enrichment profile is normalized to 1. Mouse BMAL1 bHLH includes residues 73–135 and mouse CLOCK bHLH includes residues 29–89.

The CLOCK-BMAL1 structure at SHL−6.2 wedges the entire bHLH fold between the DNA duplex and histones H2A and H3, juxtaposing the bHLH loop of BMAL1 to histone H2A L2 (Fig. 2c and Extended Data Fig. 4a). Readout of this histone-facing E-box required a larger amplitude of DNA release (up to 33°), with the BMAL1 bHLH domain (for example, BMAL1 Arg^114^) substituting for some of the nucleosomal DNA–histone contacts (for example, H2A Arg^77^) (Fig. 2c). At SHL+5.8 versus SHL−6.2, the CLOCK-BMAL1 bHLH domains differ in orientation (around 90°) relative to one another. It is now the basic helix of CLOCK that is solvent-exposed (compare Fig. 2a and Fig. 2c). Notwithstanding a change in bHLH orientation relative to the nucleosome, the CLOCK-BMAL1 PAS domains remain positioned atop the nucleosome disc at SHL−6.2, supported by the flexibility of the bHLH-PAS linkers^29^ (Extended Data Fig. 4b). In contrast to the SHL+5.8 structure, histone interactions now involve both CLOCK and BMAL1 (Fig. 2d) through a more extensive (around 1,700 Å^2^) interface with histones H2B and H3. This model, supported by rigid-body docking and XL-MS (Extended Data Fig. 4c and Supplementary Table 2), highlights electrostatic interactions between BMAL1 residues Gln^385^ and histone H4 Glu^52^. Moreover, a conserved arginine (Arg^173^) within the BMAL1 PAS-A domain is positioned adjacent to the negatively charged H2A Asp^72^ and the dipole of the H2A α2-helix (Fig. 2f). Mutation of BMAL1 Arg^173^ and Gln^385^ to alanine, accordingly, resulted in diminished nucleosome binding in lanthanide chelate excite time-resolved fluorescence resonance energy transfer (LANCE TR-FRET) experiments (hereafter, TR-FRET), while not affecting free DNA binding (Extended Data Fig. 4d–i). CLOCK-BMAL1 at the histone-facing E-box (SHL−6.2) differs from the solvent-facing E-box (SHL+5.8) in the extent of DNA release and the detailed histone contacts.

The CLOCK and BMAL1 PAS-A/B domains cover the H2A–H2B acidic patch at SHL−6.2 more extensively than observed at SHL+5.8. Similarly, competition is expected with CRY1 and CRY2 for HI loop binding and with dual acidic patch binders such as BAF for nucleosomes. The acidic patch is also involved in higher-order chromatin formation by binding the H4 tail of a neighbouring nucleosome^35^. Analogous to other reported TFs^36^, nucleosome binding by CLOCK-BMAL1 at SHL−6.2 or SHL+5.8 is also expected to affect the overall chromatin architecture.

## PAS domains influence site selection

To examine the role of the observed histone–PAS interactions on CLOCK-BMAL1 E-box accessibility, we performed SeEN-seq with the E-box NCP pool and a CLOCK-BMAL1 variant that lacked the PAS domains (CLOCK-BMAL1^bHLH^). When comparing relative peak profiles between CLOCK-BMAL1^bHLH-PASAB^ and CLOCK-BMAL1^bHLH^, we found that deletion of the PAS domains changes relative access to sites around SHL−6.5 to SHL−5.5 (Extended Data Fig. 4j). Compared to the PAS-containing CLOCK-BMAL1, MYC-MAX carries a rigid LZ dimerization module. Thus, CLOCK-BMAL1^bHLH^ is structurally more similar to MYC-MAX and, notably, also has a similar SeEN-seq profile (Extended Data Fig. 4k), which suggests that the bHLH dimerization domain affects histone access.

## Histone interactions differ for bHLH TFs

**Fig. 3:**
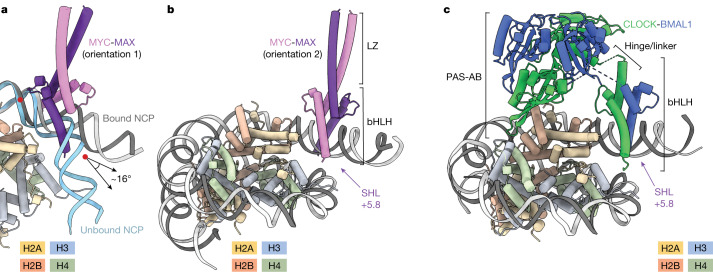
MYC-MAX bound at a solvent-exposed E-box releases DNA to accommodate its bHLH domain. **a**, Comparison of the DNA trajectory of an unbound (light blue) nucleosome to an MYC-MAX-bound (grey) nucleosome. **b**,**c**, Overall model of MYC-MAX bound to a nucleosome at SHL−6.2 (**b**) as compared to the model CLOCK-BMAL1 (**c**). MYC-MAX engages its motif without histone contacts, whereas CLOCK-BMAL1 interacts with histones H2B, H3 and H4 through the CLOCK PAS-B domain.

**Extended Data Fig. 5:**
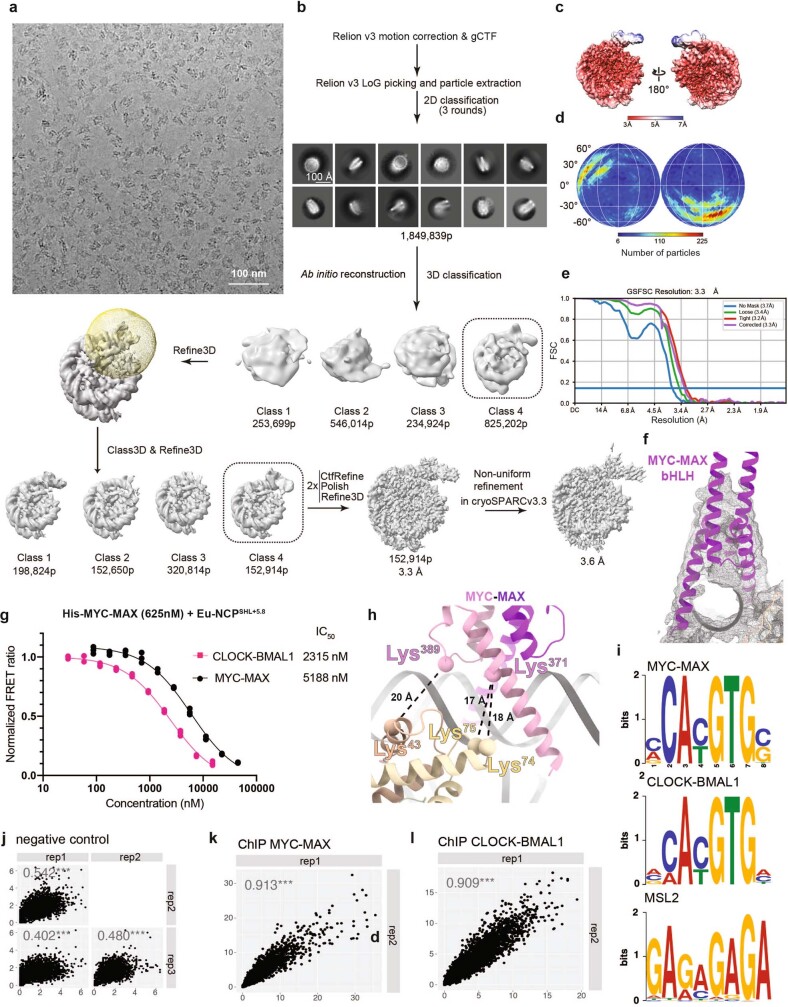
MYC-MAX and CLOCK-BMAL1 bind in proximity to nucleosomes. **a**, Representative cryo-EM micrograph of 8,841 total, denoised with Janni^81^. **b**, The movies were motion-corrected in RELION and the particles were picked using LoG picking (RELION). Multiple rounds of 2D and 3D classification (RELION) yielded a homogeneous subset of particles used for the final 3D reconstruction. The boxes defined by a dashed line indicate the good models and set of particles used for the following step in the data processing workflow. **c**, Local-resolution filtered map (MonoRes) for the 3.3 Å resolution map^90^. **d**, Angular distribution for the particles leading to the 3.3 Å resolution map. **e**, Gold-standard FSC curve for the final 3.3 Å resolution map. **f**, Map density around MYC-MAX at position SHL+5.8, contoured at 0.0948 (map postprocessed by LocScale)^88^. **g**, CLOCK-BMAL1 binds NCP^SHL+5.8^ with higher affinity than MYC-MAX. TR-FRET counter-titration of unlabelled CLOCK-BMAL1 or MYC-MAX into the preassembled Eu-NCP^SHL+5.8^-His-MYC-MAX complex. Three technical replicates are shown for each condition and three biological replicates were performed with similar results. **h**, Cross-links between MYC and H2A and H2B lysines (spheres). The cross-linker was DSSO and indicated distances (dashes) are between lysine Cα atoms. **i**, Position weight matrices (PWMs) of the binding motifs found within the peaks of each ChIP–seq profile as determined by MEME motif discovery (-mod anr -dna -revcom). **j**–**l**, Replication correlation analysis for the ChIP–seq samples used in Fig. 4c. The *D. melanogaster* genome (dm6) was queried for 5,000 hits of the E-box motif CACGTG. Read counts at each motif were normalized, counted for each replicate and replicates were compared in scatter plots. The correlation coefficients are indicated with two-tailed Pearson *P* values annotated at *P* < 0.1 (*), 0.05 (**) and 0.01 (***).

To directly examine differences and similarities between bHLH-PAS and bHLH-LZ proteins, we determined the structure of MYC-MAX bound to a nucleosome substrate identical to that used for CLOCK-BMAL1 with a solvent-exposed E-box at SHL+5.8 (MYC-MAX-NCP^SHL+5.8^). A cryo-EM envelope with an overall resolution of 3.3 Å positioned the bHLH moiety (local resolution of 4-6Å) similarly to that previously observed in the corresponding CLOCK-BMAL1 structure (Fig. 3a and Extended Data Fig. 5a–e). Unlike CLOCK-BMAL1, MYC-MAX does not contain flexible linkers adjoining bHLH and dimerization domains. Its LZ directly extends from the bHLH domain towards the solvent (Fig. 3b,c), where it does not interact with the histones (Extended Data Fig. 5f). Although the DNA-binding mode and orientation of the bHLH domain are shared between MYC-MAX and CLOCK-BMAL1, both complexes differ in their histone interactions mediated by the dimerization domain. Accordingly, the relative affinities for NCP^SHL+5.8^ in TR-FRET counter titrations are higher for CLOCK-BMAL1 than for MYC-MAX (Extended Data Fig. 5g).

The palindromic E-box allows MYC-MAX binding in two orientations, with either MYC or MAX facing the nucleosome. XL-MS identified cross-links between MYC and both H2A and H2B (Extended Data Fig. 5h and Supplementary Table 2), consistent with MYC at the histone interface.

## bHLH TFs bind E-boxes close to histones

**Fig. 4:**
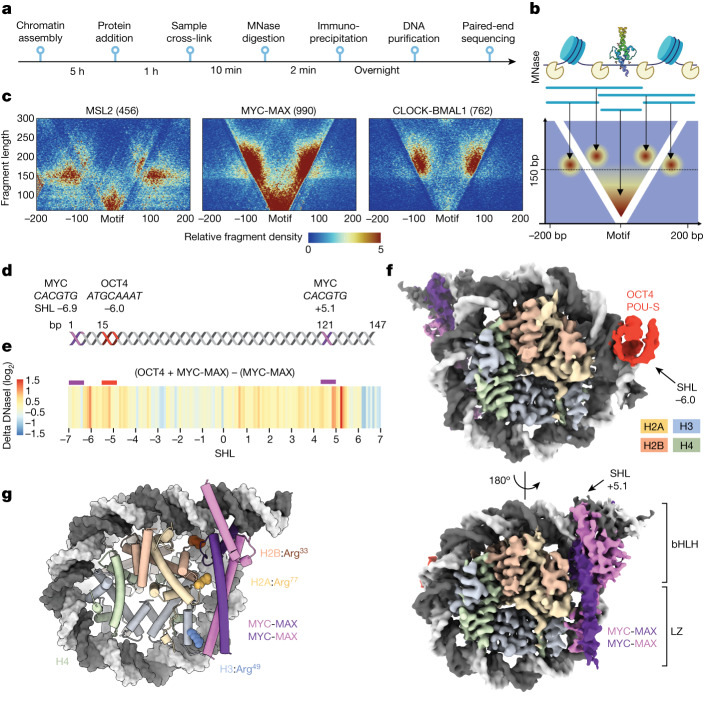
OCT4 facilitates MYC-MAX access at internal positions. **a**, Timeline of the experiment. **b**, Schematic for interpretation of V-plots. Each dot represents a sequencing read where the location of its midpoint is plotted in relation to the motif (*x* axis) and its size (*y* axis). The graph cumulates all reads from each peak and represents them as a colour-coded density plot. **c**, V-plots of ChIP–seq experiments centred to their binding motif. Fragment sizes are plotted relative to their location around the motif. Numbers in brackets indicate the number of binding sites scored in each experiment. **d**, Schematic representation of the DNA sequence, containing two E-box sites (purple) and one OCT4 site (red). **e**, The difference in DNaseI digestion across the nucleosome, in the presence of OCT4 and MYC-MAX as compared to MYC-MAX alone. **f**, Cryo-EM map of OCT4 and MYC-MAX at a resolution of 3.8 Å. **g**, Model of MYC-MAX bound at SHL+5.1. Histone arginine residues (shown as spheres) engage DNA in the uncomplexed canonical nucleosome structure^28^.

The contacts between bHLH TFs and histones suggest that these TFs have a functional role in the selection of E-box sites in chromatin. To test this hypothesis in a system without predefined nucleosome positions, we reconstituted chromatin from extracts of *Drosophila melanogaster* preblastoderm embryos (DREX). Incubation of the extracts with the corresponding genomic DNA in the presence of ATP establishes a dynamic chromatin template with physiological nucleosome spacing through the action of chromatin remodellers and histone chaperones^37^. The DNA template used contains around 33,500 CACGTG E-box motifs, allowing examination of the binding of exogenously added TFs (for example, MYC-MAX or CLOCK-BMAL1) in large excess compared to the trace amounts of endogenous TFs present in the extract^38^. Chromatin was assembled, after which MYC-MAX or CLOCK-BMAL1 were added, followed by cross-linking. After micrococcal nuclease (MNase) digestion, the TF-binding profile was analysed by chromatin immunoprecipitation with sequencing (ChIP–seq) (Fig. 4a). In total, 762 and 990 peaks were called in ChIP–seq for CLOCK-BMAL1 and MYC-MAX, respectively. MEME motif enrichment analysis yielded canonical E-box motifs in all profiles, confirming the selective binding of CLOCK-BMAL1 and MYC-MAX to the motif used in our structural studies (Extended Data Fig. 5i). Plotting the MNase fragment length against the distance from the E-boxes yields characteristic V-plots^39^ (Fig. 4b). In this analysis, the fragment sizes inform about the position of the TF relative to neighbouring nucleosomes. In cases in which MNase cannot cleave between the bound TF and a proximal nucleosome, the resulting fragments are larger than 150 bp and reside within the two arms of the ‘V’.

**Extended Data Fig. 6:**
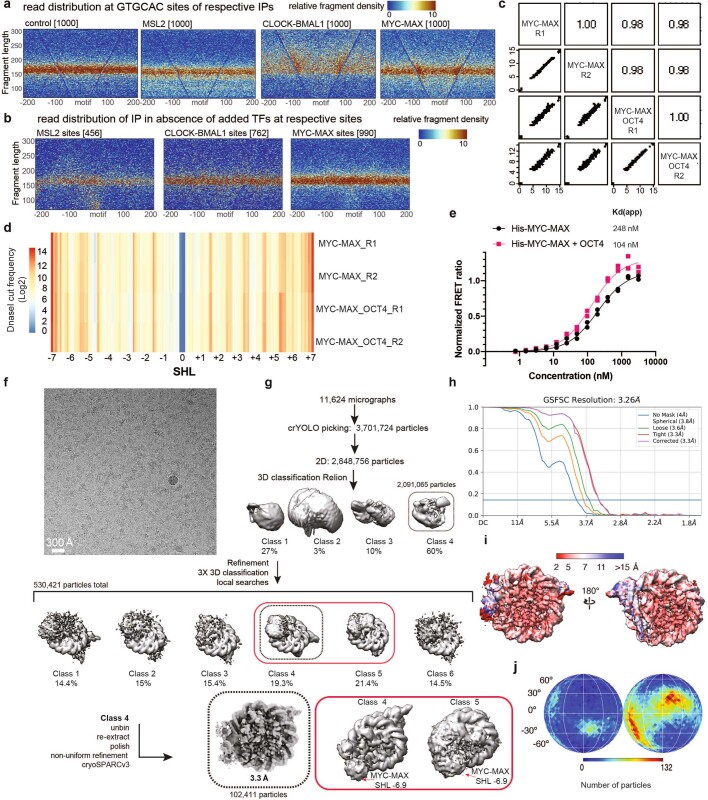
OCT4 facilitates MYC-MAX binding at SHL+5.1. **a**, Controls for non-specific effects of added TFs. V-plots of ChIP–seq experiments of the α-SpyTag control (no protein added), MSL2, CLOCK-BMAL1 and MYC-MAX centred at the reverse motif (GTGCAC). Fragment sizes are plotted relative to their location around 1,000 randomly chosen genomic motifs. The thin V-shape originates from the protection of these sites by an unknown protein present in DREX. **b**, Fragment distributions at E-box motifs analysed in Fig. 4c in the absence of added TFs. V-plots of ChIP–seq experiments with the α-SpyTag without added TFs at the peaks called in the respective IPs (see Fig. 4c). Fragment sizes are plotted relative to their location around the motif. Numbers in brackets indicate the number of binding sites scored in each experiment. **c**, Pairwise correlations of DNaseI measurements, separated by protein condition. **d**, DNaseI digestion profile across nucleosomes in the presence of MYC-MAX or MYC-MAX and OCT4. Two replicates are shown. **e**, Comparison of His-MYC-MAX binding to NCP^SHL+5.1^ in the presence and absence of OCT4. Incubation of biotinylated NCPs with LANCE Eu-W8044 streptavidin (donor) with increasing amounts of His–MYC-MAX bound by an Ultra Light α-6×His antibody (acceptor) in the presence or absence of OCT4. Two representative technical replicates are shown for each condition, and four biological replicates were performed with similar results. The signal was corrected for direct acceptor excitation by subtracting the signal observed in the absence of the nucleosome. The resulting raw signals were normalized to the individual Bmax values, and binding curves were fit using a one-site specific binding model. **f**, Representative cryo-EM denoised with Janni of 11,624 total micrographs. **g**, See Methods. The movies were pre-processed within cryoFLARE and the particles were picked using crYOLO^81^. Multiple rounds of 3D classification yielded a homogeneous subset of particles that were used for the final 3D reconstruction. The boxes defined by a dashed line indicate the good models and set of particles used for the following step in the data processing workflow. **h**, Gold-standard FSC curve for the 3.3 Å resolution map highlighted by the dashed box shown in **c**. **i**, Local-resolution filtered map (MonoRes) for the 3.3 Å resolution map. The highest resolution was found around the NCP ranging from 2–5 Å, whereas for OCT4 and MYC-MAX the resolution ranged between 5 Å and 11 Å. **j**, Angular distribution for the particles leading to the 3.3 Å resolution map.

Nucleosome–TF signatures inside the ‘V’ were observed for CLOCK-BMAL1 and MYC-MAX, indicating TF binding proximal to nucleosomes (Fig. 4c). The V-profiles obtained were in stark contrast to the *Drosophila* TF MSL2 (Fig. 4c), in which short reads within the ‘V’ and centred around the motif represent the binding of TFs to accessible linker DNA. Fragments of 150 bp or longer outside the ‘V’ indicate phased nucleosomes separated from the motif (Fig. 4c). For CLOCK-BMAL1, almost no short fragments were mapped. Instead, most motif-containing fragments were larger and clustered in groups of about 180 bp in length within the V-arms, with the motif 80 bp up- and downstream of the centre of the read. These fragments therefore originate from cleavage events on either side of a nucleosome, with CLOCK-BMAL1 bound to an E-box at or near the entry–exit site, consistent with the positional preference seen in SeEN-seq and the corresponding structures (Figs. 1b,f and 2d). In DREX, nucleosomes are not particularly pre-positioned around E-boxes without TFs (Extended Data Figs. 5j–l and 6a). Yet, when comparing CLOCK-BMAL1 V-plots (Fig. 4c) to those of ‘classical’ TFs^38^, we find E-boxes with CLOCK-BMAL1 residing immediately adjacent to the histone octamer. These effects are specific to E-boxes, as an inverted or scrambled E-box motif shows no nucleosome positioning (Extended Data Fig. 6b).

MYC-MAX binding yields a V-plot with signatures similar to CLOCK-BMAL1 (Fig. 4c), indicating that other E-box binders can also position nucleosomes. The analysis shows small fragments (shorter than 100 bp) around the motif originating from isolated MYC-MAX binding to linker DNA. Notably, the fragment distribution inside the ‘V’ shows a continuum of sizes between 110 bp and 140 bp; these fragments originate from a juxtaposed nucleosome, yet are more subnucleosomal. A possible explanation is that MYC-MAX can bind internal E-boxes facilitated by extensive DNA unwrapping from the nucleosome.

## MYC and OCT4 cooperate on nucleosomes

In cell-fate determination and differentiation, MYC operates with the other Yamanaka factors OCT4, SOX2 and KLF4 (ref. ^40^). OCT4 has also been reported to work in concert with MYC-MAX to assist binding at chromatinized motifs in cells^41^. We first tested whether the cooperative action between OCT4 and MYC-MAX would allow binding at more internal sites. Therefore, we constructed a nucleosome with an E-box at a solvent-facing position (SHL−6.9) highly enriched for MYC-MAX binding in our SeEN-seq assay (Fig. 1c), together with an additional OCT4 site (SHL−6.0) downstream of this E-box, maintaining a second more internal E-box at SHL+5.1 from the original W601 template (Fig. 4d). DNaseI footprinting experiments indicated that MYC binding at SHL+5.1 is enhanced by OCT4, as evident by the emergence of a DNaseI hypersensitive site near SHL+5.1 (Fig. 4e and Extended Data Fig. 6c,d). To directly measure the effect of OCT4 on MYC-MAX engagement, we used a TR-FRET assay in which His–MYC-MAX was added to biotinylated nucleosomes in the presence of the FRET pairs, LANCE Eu-W8044 streptavidin and Ultra ULight α-6×His antibody^42^. The binding isotherms of MYC-MAX to nucleosomes were strengthened by around threefold in the presence of OCT4 (Extended Data Fig. 6e). Hence, OCT4 binding facilitates MYC-MAX engagement across the dyad at an internal motif position.

## The MYC-MAX LZ binds to histones

**Extended Data Fig. 7:**
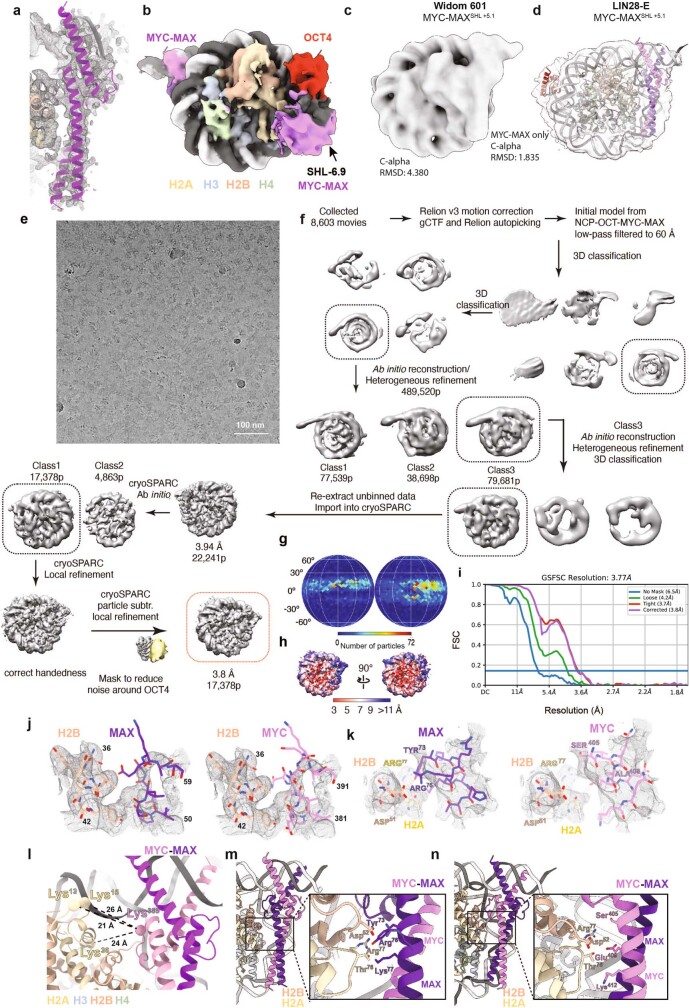
MYC-MAX and OCT4 cooperatively bind to a nucleosome by releasing nucleosomal DNA. **a**, Map density around MYC-MAX at position SHL+5.1, contoured at 0.0121 (map postprocessed by LocScale). **b**, A second diffuse MYC-MAX heterodimer is present in some classes (see also Extended Data Fig. 6g) at SHL-6.9. **c**,**d**, Comparison of OCT4–MYC-MAX-Widom 601 (**c**) and the OCT4–MYC-MAX-LIN28-E nucleosome (**d**) complexes. **e**, Representative cryo-EM micrograph of 8,603 micrographs, denoised with Janni. **f**, Processing scheme. The movies were pre-processed with cryoFLARE and the resulting movies were imported in RELION for motion correction, CTF estimation and particle picking. Ab-initio (cryoSPARC) in combination with 3D classification (RELION) resulted in a homogenous subset of particles that were used for the final 3D reconstruction. The boxes defined by dashed line indicate the good models and set of particles used for the following step in the data processing workflow. **g**, Angular distribution for the particles leading to the 3.8 Å resolution map. **h**, Local-resolution filtered map (MonoRes) highlighted by red dashed box shown in **f**. **i**, Gold-standard FSC curve for the 3.8 Å resolution map highlighted by the red dashed box shown in **f**. **j**, Map density around the interface between the basic loop of MYC or MAX and H2B, contoured at 0.13. **k**, Map density around a contact between MYC or MAX and H2B/H2A, contoured at 0.096. Maps were postprocessed by LocScale^88^. Residues Tyr^73^ and Arg^76^ in MAX were mutated to Ala and residues Ser^405^ and Ala^408^ in MYC were mutated to Tyr and Arg, respectively to mimic the residues in MAX, making MYC more MAX-like for smTIRF experiments (see also Extended Data Fig. 8a–n). **l**, Cross-link between MYC basic loop and H2A lysines (spheres). The cross-linker was DSSO and indicated distances (dashes) are between lysine Cα atoms. **m**,**n**, Close-up of the TF–histone interface for both MYC-MAX orientations, highlighting potentially contacting residues between H2A/H2B and the LZ. Side-chain rotamers, shown here, are modelled, as clear density was missing.

To examine how MYC-MAX accesses this internal, histone-facing E-box at SHL+5.1 together with OCT4, we determined a 3.3-Å structure of the nucleosome complex bound to OCT4 and MYC-MAX with a local resolution of MYC-MAX of 4-11 Å (Extended Data Figs. 6f–j and 7a). We found that OCT4 engaged with DNA only through its POU-specific (POU-S) domain, leading to the release of DNA from the histone octamer over 14 bp (Fig. 4f), similar to what has previously been observed^25^. On the other end of the nucleosome, we detected MYC-MAX bound to the internal E-box at SHL+5.1 (Fig. 4f). In two three-dimensional (3D) classes, a diffuse density for a second MYC-MAX dimer at the entry–exit site (SHL–6.9) adjacent to OCT4 was observed (Extended Data Figs. 6g and 7b). This dimer was distal from the histones and not sufficiently ordered to allow structure determination. Instead, we observed that the MYC-MAX dimer at SHL+5.1 engaged in extended interactions with histones H2B (around 280 Å^2^), H2A (around 180 Å^2^) and H3 (around 100 Å^2^), concomitant with an approximately 30-bp release of DNA from the nucleosome. The MYC-MAX bHLH-LZ fold covers large parts of the histones H2A, H2B and H3 surface orphaned by DNA release. The arginine anchor residues contacting the minor groove of the wrapped nucleosomal DNA (for example, H2A:Arg^77^ at SHL+5.5) are repurposed in the presence of MYC-MAX to engage the LZ (Fig. 4g). We also determined the structure of a highly analogous MYC-MAX and OCT4 nucleosome complex using the endogenous Lin28-derived nucleosome DNA sequence (LIN28-E) with added motifs for MYC-MAX (SHL+5.1) and OCT4 (SHL−6.0) (3.8 Å overall, 6–11 Å for MYC-MAX) (Extended Data Fig. 7c,d). The structures were similar, suggesting that the MYC-MAX binding mode is independent of the nucleosome backbone used (Extended Data Fig. 7e–i). The approximately 30-bp DNA release in the W601 and Lin28-E structures after MYC-MAX binding at SHL+5.1 would also result in subnucleosomal MNase fragments, consistent with the V-plot analysis of the chromatin reconstitutions (Fig. 4c).

OCT4 and MYC-MAX are not engaging in protein–protein interactions, and the additive effect of OCT4 on facilitating MYC-MAX binding is therefore indirect. The increased overall destabilization of the nucleosomal DNA structure by OCT4 in DNaseI experiments (Fig. 4e), in conjunction with the extensive peeling off of the DNA, suggests a mechanism in which OCT4 primes nucleosomal templates for the required DNA distortions to accommodate MYC-MAX at an internal site.

**Extended Data Fig. 8:**
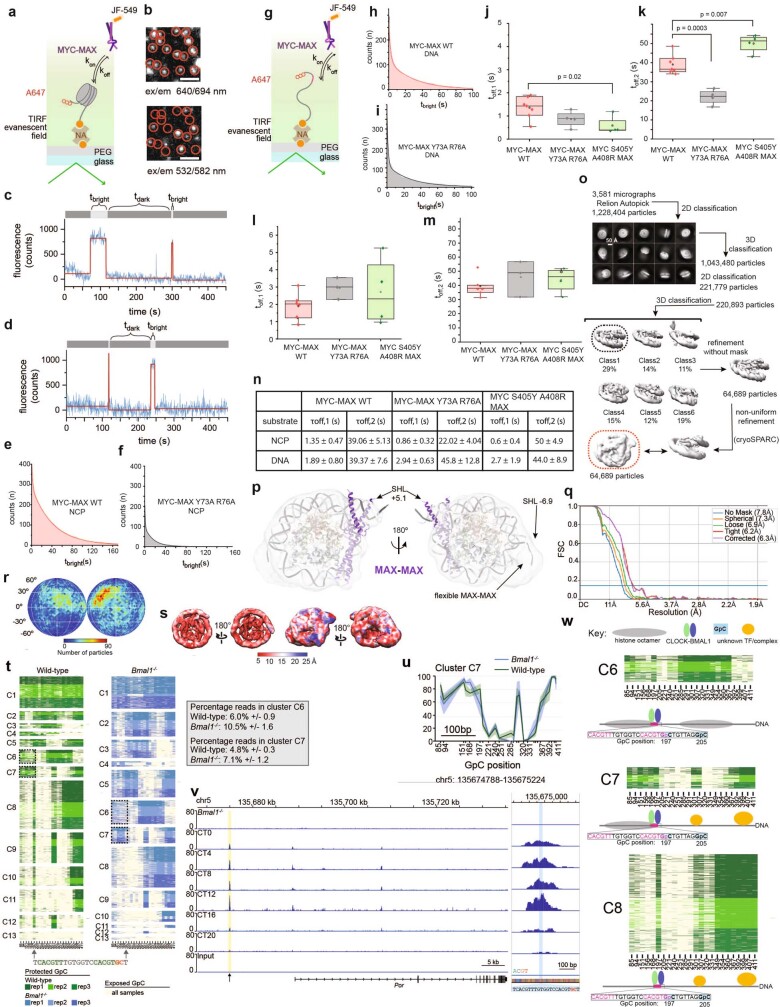
TF–histone contacts are relevant in vitro and in vivo. **a**, Scheme of the experiment: MYC-MAX (WT or mutant), labelled with JF549, is injected into flow cells containing immobilized Alexa647-labelled NCPs. Dynamic MYC-MAX binding events are detected by colocalization single-molecule (sm) TIRF imaging. **b**, Detection of DNA or nucleosome (NCP) localizations using smTIRFM in 640/694 nm channel and single MYC-MAX binding events are detected at DNA positions by smTIRFM in 532/582 nm channel, through a colocalization algorithm. Scale bars: 2 μm. The images are representative of 3 independent experiments. The statistical details for each experiment are listed with the quantification of the signal. **c**, Extracted fluorescence time trace for 2 nM MYC-MAX WT, showing stochastic binding events to NCPs. **d**, Fluorescence time trace for MYC-MAX^Y73A,R76A^ binding to NCPs. **e**, Dwell-time histogram for MYC-MAX WT binding to NCPs. For fit results, yielding two dwell times (*τ*off,1; *τ*off,2) see j, k. **f**, Dwell-time histogram for MYC-MAX^Y73A,R76A^ binding NCPs. For fit results, yielding two dwell times (*τ*_off,1_; *τ*_off,2_) see **j**,**k**. **g**, Scheme of the experiment: MYC-MAX (WT or mutant) with Alexa647-labelled DNA. **h**, Dwell-time histogram for MYC-MAX WT binding to DNA. For fit results, yielding two dwell times (*τ*_off,1_; *τ*_off,2_) see **l**,**m**. **i**, Dwell-time histogram for MYC-MAX^Y76A,R73A^ binding to DNA. For fit results, yielding two dwell times (*τ*_off,1_; *τ*_off,2_) see **l**,**m**. **j**,**k**, Dwell times (*τ*_off,1_; *τ*_off,2_) for MYC-MAX WT, MYC-MAX^Y73A,R76A^ and MYC^S405Y,A408R^-MAX binding to NCPs. The indicated numbers are *P* values (two-tailed Student’s *t*-test, with *n* = 4 (MYC-MAX^Y73A,R76A^), 7 (MYC-MAX WT) and 4 (MYC^S405Y,A408R^-MAX) ([independent experiments]). **l**,**m**, Dwell times (*τ*_off,1_; *τ*_off,2_) for MYC-MAX WT, MYC-MAX^Y73A,R76A^ and MYC^S405Y,A408R^-MAX binding to DNA. The indicated numbers are *P* values (two-tailed Student’s *t*-test, with *n* = 3 (MYC-MAX^Y73A,R76A^), 6 (MYC-MAX WT) and 3 (MYC^S405Y,A408R^-MAX) ([independent experiments]). In **j**–**m** the bottom of the boxes defines the first quartile (Q1 or 25th percentile), the middle indicates the median (Q2 or 50th percentile), and the top the third quartile of the data (Q3 or 75th percentile). Whiskers are extended up to the most extreme data point that is no more than 1.5 × IQR. All data points are shown for each box with a mean shown in white. **n**, Dwell times for MYC-MAX proteins binding to the different substrates. **o**, The movies were pre-processed with cryoFLARE and the resulting movies were imported in RELION for particle picking. Multiple rounds of 2D and 3D classification (RELION) resulted in a homogenous subset of particles used for the final 3D reconstruction. The boxes defined by dashed line indicate the good models and set of particles used for the following step in the data processing workflow. **p**, Overlay of the cryo-EM map of the MAX-MAX- (at SHL+5.1 and SHL−6.9) bound nucleosome and the model showing MAX-MAX bound at SHL+5.1. **q**, Gold-standard FSC curve for the 7 Å resolution map highlighted by the red dashed box shown in **o**. **r**, Angular distribution for the particles leading to the 7 Å resolution map. **s**, Local-resolution filtered map (MonoRes) highlighted by red dashed box shown in **o**. **t**, DNA protection analysis at a CLOCK-BMAL1 enhancer by SMF. SMF was performed in mouse liver at a distal enhancer of the gene *Por* (chr. 5:135674788–135675224). Heat maps displaying protection from GpC methylation on each single DNA molecules at that enhancer, with unprotected/methylated cytosines coloured in yellow, and protected/unmethylated cytosines coloured in green (WT mouse at zeitgeber time (ZT) 6 or blue (Bmal1^−/−^ at ZT6). Shades of green and blue distinguish three biological replicates for each group. Reads from all 6 animals (n = 1,052 reads per sample) were clustered by the Binary Matrix Decomposition clustering algorithm in a total of 13 clusters. Each column illustrates protection at a single GpC, spanning 327 bp. The arrows at the bottom of the heat maps point to a GpC in a CLOCK-BMAL1 DNA-binding motif (E-box sequence shaded in green). The dashed boxes in clusters C6 and C7 indicate an enhanced protection region immediately upstream of a CLOCK-BMAL1 binding motif, suggesting protection by a nucleosome. For sequencing reads see Supplementary Table 3. Quantification of the percentage of reads ± s.e.m. in clusters C6 and C7 for both wild-type and Bmal1^−/−^ mice. **u**, The graph displays the percentage of protection at each GpC for cluster C7, with the lines and shaded area representing the average ± s.e.m. of three biological replicates for wild-type (green) and *Bmal1*^−/−^ (blue) mice. **v**, Genome browser view of BMAL1 ChIP–seq signal at *Por* gene locus in mouse liver. Sequencing data were retrieved from GSE39860^21^. The arrow and yellow-shaded area point to the distal enhancer analysed by SMF. Zoom in the whole amplicon analysed by SMF (chr5:135674788–135675224), with the blue area indicating the location of CLOCK-BMAL1 DNA-binding motif. **w**, Schematic representation of predicted DNA-bound proteins corresponding to the observed footprints.

The MAX LZ facing the histones best accounts for the detailed density envelope for MYC-MAX (model map correlation, 0.59). However, the assignment is not unambiguous, given the symmetric E-box motif and the structural similarity between MYC and MAX (Extended Data Fig. 7j,k). In XL-MS, a single cross-link between MYC and histone H2A was identified and is best explained by MYC facing histone H2A (Extended Data Fig. 7l and Supplementary Table 2). On the other hand, measurements with wild-type MAX and mutants in single-molecule total internal reflection fluorescence microscopy (smTIRFM) with a nucleosome containing a single canonical E-box at SHL+5.1 implicated MAX residues Tyr^73^ and Arg^76^ at the histone interface (Extended Data Figs. 7m,n and 8a–n). Together, the data are consistent with MYC-MAX binding histones in both orientations through a dynamic equilibrium. A MAX-MAX homodimer may thus also be accommodated at the histone interface if MAX can engage histones. Accordingly, we determined the structure of a MAX-MAX homodimer by cryo-EM bound to a nucleosome at SHL+5.1 (6.2 Å overall; 10–15 Å for MAX-MAX). After low-pass filtering to equal resolutions, this gave a map similar to MYC-MAX (Extended Data Fig. 8o–s). MYC and MAX can thus be accommodated facing the histones, and other MAX dimerization partners such as MXD1–MXD4, MNT and MGA are also expected to be compatible with nucleosome binding at internal sites.

## CLOCK-BMAL1 binds entry–exit sites in vivo

The synthetic nucleosome-positioning sequences used pose the question of whether the structural and functional relationships observed reflect the in vivo situation. Analogous to MYC-MAX and OCT4 binding at the W601 versus the endogenous Lin28-E, we sought to determine how CLOCK-BMAL1 binds to native nucleosome backbones.

**Fig. 5:**
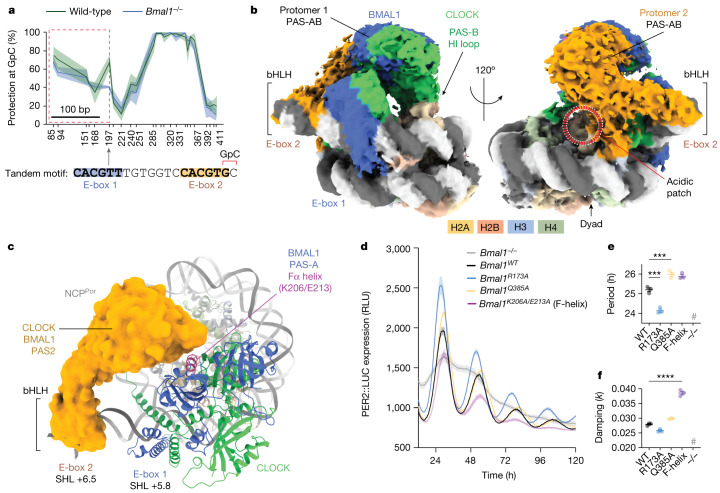
CLOCK-BMAL1 uses protein–protein interactions to engage an endogenous locus. **a**, SMF was performed in mouse liver at the *Por* gene (chr. 5: 135674788–135675224). Sequencing reads were clustered on the basis of DNA protection profiles at every GpC. Two clusters (C6 and C7) showed increased DNA protection of a nucleosome-sized fragment encompassing tandem E-boxes targeted by CLOCK-BMAL1 (see also Extended Data Fig. 8t–w). The graph shows the percentage of protection at each GpC for cluster C6, with the lines and shaded area representing the average ± s.e.m. of three biological replicates for wild-type (green) and *Bmal1*^−/−^ (blue) mice. The grey arrow at position 197 points to the GpC directly downstream of E-box 2, marked in red. The dashed red box illustrates an increase of DNA protection over 112 bp that is suggestive of a nucleosome and is the DNA sequence used for cryo-EM. **b**, Cryo-EM map of the *Por* nucleosome-bound by two protomers of CLOCK-BMAL1. **c**, The BMAL1 PAS-A Fα helix of the internal protomer (E-box 1) interfaces with the PAS domains of the external protomer at E-box 2. The E-box 2 protomer is depicted as a cryo-EM map segment (Segger, ChimeraX)^56^. **d**–**f**, PER2::LUC expression from *Bmal1*^−/−^ Per2::Luc mouse fibroblast cells stably reconstituted with wild-type (WT) or mutant *Bmal1* (**d**), presented as relative light units (RLU). There are significant differences in period (**e**) and damping (**f**) of the PER2 oscillation. *n* = 3 biological replicates, mean ± s.e.m. One-way ANOVA, Dunnett’s multiple comparisons test (two sided). *P ≤ 0.05, **P ≤ 0.01, ***P ≤ 0.001, ****P ≤ 0.0001. For period analysis (**e**), WT versus R173A is *P* = 0.0001, and WT versus Q385A is *P* = 0.0009. For damping analysis (**f**), WT versus F-helix is *P* < 0.0001.

**Extended Data Fig. 9:**
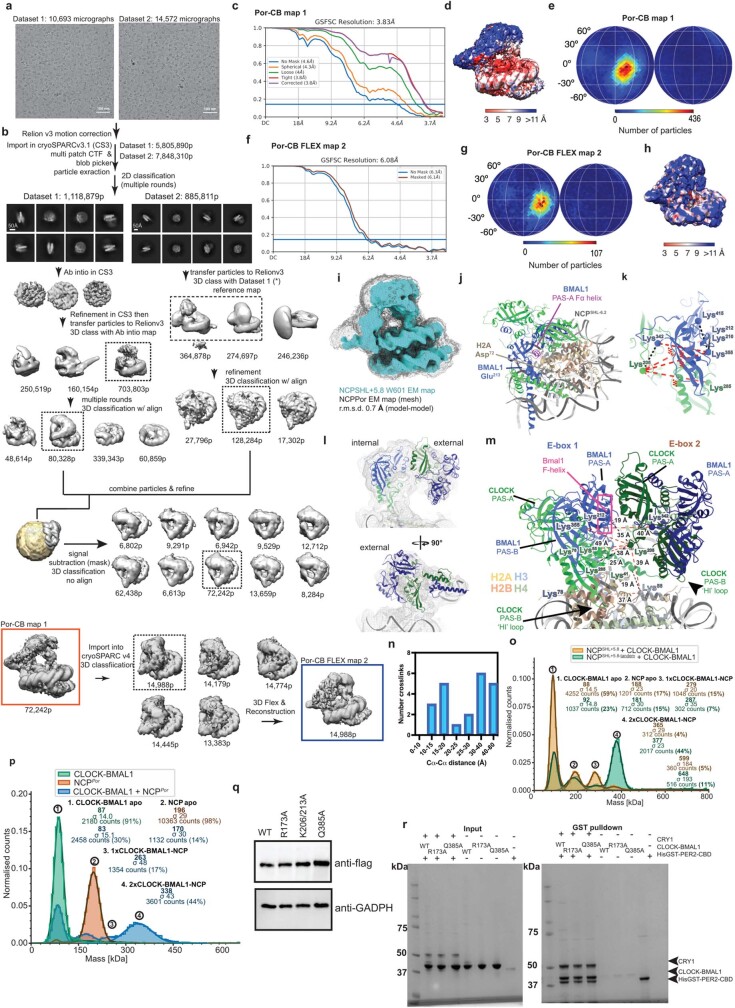
CLOCK-BMAL1 engages in protein–protein interactions on tandem E-boxes. **a**, Representative cryo-EM micrographs from two collected datasets (10,693 micrographs, dataset 1; 14,572 micrographs, dataset 2) denoised with Janni. **b**,. Movies were motion-corrected in RELION v.3, then CTF correction, particle picking as well as multiple rounds of 2D classification were performed in cryoSPARC v.3.1. Particles from dataset 1 were used for 3D reconstruction and after refinement, were transferred into RELION. They were used as an input model for 3D classification of dataset 2 in RELION. After multiple rounds of 3D classification and refinement both datasets were merged and subsequent 3D classification with signal subtraction and 3D Flex reconstruction yielded a homogeneous subset of particles. The boxes defined by the dashed line indicate the good models and set of particles used for the following step in the data processing workflow. **c**, Gold-standard FSC curve for the 3.8 Å resolution map highlighted by the red box in **b**. **d**, Local-resolution filtered map (MonoRes) for the 3.8 Å resolution map highlighted by the red box shown in **b**. **e**, Angular distribution for the particles leading to the 3.8 Å resolution map. **f**, Gold-standard FSC curve for the 6.1 Å resolution map highlighted by the blue box shown in **b**. **g**, Angular distribution for the particles leading to the 6.1 Å resolution map. **h**, Local-resolution filtered map (MonoRes) for the 6.1 Å resolution map highlighted by the blue box shown in **b**. **i**, Internal CLOCK-BMAL1 in *Por* map overlays well with the single CLOCK-BMAL1 heterodimer bound in the NCP^SHL+5.8^-W601 structure. **j**, F-alpha PAS-A helix of BMAL1 interfaces with the histones when CLOCK-BMAL1 binds at SHL-6.2. **k**, Sterically incompatible cross-links when mapped to the PAS domains of a single CLOCK-BMAL1 heterodimer. **l**, Map fit of tentative tandem CLOCK-BMAL1 model best compatible with cross-linking and cryo-EM data. The map is at 0.005. **m**, Tentative CLOCK-BMAL1 tandem model with putative inter-CLOCK-BMAL1 and CLOCK-BMAL1-histone cross-links mapped. Putative inter-CLOCK-BMAL1 cross-links would be sterically incompatible when mapped to a single heterodimer (see **k**). **n**, Distance distribution of cross-links mapped to the tandem CLOCK-BMAL1 model shown in panel **m**. **o**, Molecular mass distribution histogram of CLOCK-BMAL1-NCP^SHL+5.8^ (single E-box) and CLOCK-BMAL1-NCP^SHL+5.8-tandem^ (2 E-boxes with 7-bp spacing as in the *Por* structure but with a 601 sequence). The tandem E-box arrangement increased the amount of CLOCK-BMAL1-bound complex from 19% to 51%. **p**, Molecular mass distribution histogram of CLOCK-BMAL1-NCP^Por^. **q**, Western blot comparing BMAL1 protein expression across reconstituted cell lines. The blot is representative of 3 biological replicates. **r**, GST pull-down assay performed by incubating His–GST-tagged CRY-binding domain of Per2 (His–GST-PER2-CBD) as bait with the prey proteins: photolyase homology region (PHR) of CRY1 and CLOCK-BMAL1 wild-type or mutant constructs. CLOCK and BMAL1 bHLH PAS-AB both are of very similar molecular weight, therefore, appear as one single band. The gel shown is representative of *n* = 3 independent experiments.

Performing single-molecule footprinting (SMF) in the liver of wild-type and *Bmal1*^−/−^ mice, we analysed the enhancer distal to the *Por* gene, previously shown to be targeted by CLOCK-BMAL1, exhibiting rhythmic nucleosome signals^21,43^ (Extended Data Fig. 8t,u). Two clusters were identified showing DNA protection of more than 100 bp upstream of tandem E-boxes, consistent with an E-box embedded nucleosome (Fig. 5a, Extended Data Fig. 8t–w and Supplementary Table 3). Robust BMAL1 binding has previously been reported at tandem E-boxes^5,6,21,44^. Accordingly, the protection signal at this motif, with two E-boxes spaced 7 bp apart increased in wild-type mice relative to *Bmal1*^−/−^ cells (especially in cluster C6; Fig. 5a). To test whether this footprint is consistent with CLOCK-BMAL1 binding at a nucleosome-occupied locus, we used the 147-bp DNA sequence of the C6 and C7 nucleosome for reconstitution in the presence of CLOCK-BMAL1, and determined the structure by cryo-EM (Extended Data Fig. 9a–h).

The 3.8-Å structure of the endogenous *Por* sequence (NCP^*Por*^) accommodates two CLOCK-BMAL1 protomers engaging the nucleosomal ends from SHL+5.0 to SHL+6.5 in line with end-binding behaviour (Fig. 5b). The two bHLH DNA-binding domains are angled around 40° from one another. The more internal CLOCK-BMAL1 molecule (E-box 1) (local resolution 4–8 Å) superimposes well with the CLOCK-BMAL1 structure at SHL+5.8 in the W601 backbone (Extended Data Fig. 9i). Consistent with its binding preferences in SeEN-seq, CLOCK-BMAL1 enforces a solvent-exposed register of E-box 1 in the *Por* backbone (Fig. 5c). The similarity between these structures further supports the notion that the backbone sequence (endogenous versus artificial) does not substantially affect the binding mode.

Direct protein–protein interactions at tandem E-boxes between CLOCK-BMAL1 heterotetramers have previously been suggested on the basis of modelling^3,6^. We observe that the two CLOCK-BMAL1 protomers engage in extensive interactions with one another and the histone core, mediated by the PAS domains. CLOCK at E-box 1 forms well-defined interactions with the histone core, with the HI loop of the CLOCK PAS-B contacting the H3α1 L1 elbow, sterically occluding the acidic patch. The BMAL1 face of the internal heterodimer (E-box 1) mediates interactions with the external heterodimer (E-box 2). The F-α PAS-A helix of BMAL1 (residues 206–213) is central to tandem PAS–PAS interactions between CLOCK-BMAL1 protomers (Fig. 5c). The identical helix also interfaces with the histone core when CLOCK-BMAL1 engages its single E-box motif at SHL−6.2 (Extended Data Fig. 9j), highlighting the functional importance of this region. In the 3.8-Å overall structure, the local resolution, of the PAS domains of the distal protomer bound to E-box 2, is around 8–11 Å. On the basis of XL-MS and map interpretation, we provide a tentative model for E-box 2 with the PAS domains residing on top of but not interacting with the histone core (Extended Data Fig. 9k–n).

A tandem motif spacing of 6–7 bp is frequently observed in the promoters of core circadian genes^5–7^ (*Per1*, *Per2* and *Per3*), which is required for robust daily oscillations^7^. The binding of CLOCK-BMAL1 to tandem E-boxes was found to be cooperative on free DNA^5^. In mass photometry, tandem E-boxes relative to single E-boxes on nucleosomes increase- the total amount of CLOCK-BMAL1 bound from 19% to 51% (Extended Data Fig. 9o,p). The Por structure, with its tandem arrangement, thus identifies cooperative protein–protein interactions between two CLOCK-BMAL1 protomers as a further strategy to engage chromatinized E-boxes.

## TF–histone contacts have a role in transcription

To investigate the functional importance of the identified protein–protein interactions, we selectively mutated residues in *Bmal1* that formed part of the most extended interactions observed in our structures (Figs. 2 and 5) and examined the mutant protein activity within the cellular circadian oscillator. We used a Period2-luciferase (PER2::LUC) assay in which fibroblasts from arrhythmic *Bmal1*^*–/–*^*;*PER2::LUC mice are restored through lentiviral-based genetic complementation of *Bmal1* under a constitutive promoter. Wild-type *Bmal1* reconstitution establishes robust binding of CLOCK-BMAL1 to tandem E-boxes within the endogenous *Per2* promoter to drive the rhythmic accumulation of PER2::LUC protein. To test the physiological relevance of interactions observed with the BMAL1 PAS-A F-helix at the histone (NCP^SHL−6.2^) and tandem E-box PAS interface (NCP^*Por*^), we mutated two F-helix residues, BMAL1 PAS-A:Lys^206^Glu^213^ to alanine (F-helix mutant) and tested their effect on cellular rhythmicity (Fig. 5d–f and Extended Data Fig. 9q). Cells complemented with this F-helix mutant showed an increase of around 35% in the rate of amplitude damping, highlighting the role of this CLOCK-BMAL1 helix in sustaining high-amplitude, robustly rhythmic gene expression.

As seen in the structures, CLOCK-BMAL1 forms multiple interfaces with histones as a function of the motif position (Fig. 2); we focused on mutations that specifically target BMAL1–histone interactions, reasoning that some of them would be sufficiently represented to cause a cellular phenotype when mutated. Mutation of residues BMAL1 PAS-A:Arg^173^ and BMAL1 PAS-B:Gln^385^ to alanine reduced binding to a nucleosomal template (E-box, SHL–6.2) without affecting histone-free DNA binding (Extended Data Fig. 4e–i) or interactions with known coregulators, PER2 or CRY1 (Extended Data Fig. 9r). Whereas BMAL1 PAS-B:Gln^Q385A^ produced an increase of around 45 min in the period of PER2::LUC expression, genetic complementation with the single point mutant, *Bmal1*^*R173A*^, showed a decrease of more than 1 h in the cellular period compared to cells complemented with wild-type *Bmal1* (Fig. 5d). These data show that CLOCK-BMAL1–histone interactions have an essential role in determining circadian period, and that histone contacts affect circadian gene expression and overall bHLH function.

## Discussion

Chromatin affects bHLH access; a bHLH DNA-binding domain engaging a nucleosome-embedded E-box is predicted to clash with the nucleosome at nearly all of the around 150 possible registers^45^ (Extended Data Fig. 2k,l). Nonetheless, CLOCK-BMAL1 binds to chromatinized target sites in the genome, leading to rhythmic nucleosome loss and increased accessibility for other TFs^43^. MYC-MAX prefers binding to sites in open, accessible chromatin^1,41,46^. However, several proteins, for example, OCT4, have been suggested to guide MYC to chromatinized binding sites during cellular reprogramming^41,47^. We herein provide the mechanistic and functional basis for nucleosomal E-box readout across two phylogenetically diverse bHLH members. MYC-MAX and CLOCK-BMAL1 have similar end-binding preferences on nucleosomal DNA in vitro and in vivo^48^ (Figs. 1d, 4c and 5a). They require DNA release when engaging motif positions throughout the nucleosome, resulting in extensive protein–protein interactions between TFs and the orphaned histones. Comparing the histone surfaces contacted by the bHLH TFs, we find that, in particular, interactions with H2Bα1 L1 and H2A L2 are shared between MAX-MAX (SHL+5,1), MYC-MAX (SHL+5.1, SHL+5.8) and CLOCK-BMAL1 (SHL+5.8, SHL−6.2)^4^. However, the detailed histone interactions differ as a function of protein and motif position and could be modulated by proximal histone modifications. Solvent-facing sites are generally more accessible than histone-facing motifs (Fig. 1b,c), which require larger amplitudes of DNA release, resulting in lower-affinity binding.

CLOCK-BMAL1 and MYC-MAX interact with and position nucleosomes in complex genome reconstitutions in vitro (Fig. 4c), where they prefer binding at the edge of nucleosomes. Whether positioning is due to bHLH TFs simultaneously contacting the motif and histones or is further asssisted by enzymatic sliding activities present in the extract is unclear. The biochemical ability to bind nucleosomes would allow bHLH TFs to act as boundary elements at open–closed transitions of the genome. Yet the fate of a given factor residing in open/closed chromatin ultimately depends on downstream processes such as chromatin remodelling and the cooperative action of TFs.

In vivo, the most transcriptionally active CLOCK-BMAL1-dependent genes have tandem E-boxes^5^. There, CLOCK-BMAL1 uses bHLH–histone contacts and works with a second CLOCK-BMAL1 protomer to drive DNA removal from the histones at an otherwise occluded site (Fig. 5a–c). The defined 7-bp spacings between E-boxes increase accessibility through direct protein–protein interactions between protomers on nucleosomes. Closely spaced E-boxes have been observed for other TFs^1^, and it is tempting to speculate that a subset of these also engages in defined protein–protein interactions. We further show that multiple nucleosome-bound motifs can cooperate without direct TF protein–protein interactions^49,50^. OCT4 at SHL−6.0, for example, assisted MYC-MAX binding at a distal site by around threefold (Fig. 4f and Extended Data Fig. 6e). We propose that the indirect cooperativity between the two TFs is due to destabilizing the nucleosomal DNA structure, thus facilitating the 30-bp DNA unwrapping required to sustain MYC-MAX binding.

We show that through histone contacts, direct interaction between TFs and long-range DNA-destabilization, bHLH TFs directly and/or indirectly drive binding to chromatinized DNA, providing a molecular and structural mechanism for theoretical and cellular models of TF binding to nucleosomes^23,49–53^.

## Methods

### Expression, purification and reconstitution of human octamer histones

Human histones were expressed and purified as described previously^57^. Lyophilized histones were mixed at equimolar ratios in 20 mM Tris-HCl (pH 7.5) buffer, containing 7 M guanidine hydrochloride and 20 mM 2-mercaptoethanol. Samples were dialysed against 10 mM Tris-HCl (pH 7.5) buffer, containing 2 M NaCl, 1 mM EDTA and 2 mM 2-mercaptoethanol. The resulting histone complexes were purified by size-exclusion chromatography (Superdex 200; GE Healthcare). For MYC-MAX TR-FRET experiments, H2B-biotinylated octamers were prepared using a T122C mutant introduced into H2B using site-directed mutagenesis. The purified H2A–H2B(T122C) complex (46 μM) was conjugated with biotin using 558 μM EZ-Link Maleimide-PEG2-Biotin (Thermo Fisher Scientific) in 10 mM Tris-HCl (pH 7.5) buffer, containing 2 M NaCl, 1 mM EDTA and 1 mM TCEP, at room temperature for 2 h. The reaction was stopped by adding 2-mercaptoethanol and the sample was then dialysed against 10 mM Tris-HCl (pH 7.5) buffer containing 2 M NaCl, 1 mM EDTA and 5 mM 2-mercaptoethanol. Reconstitutions of the H2A–H2B(T122C–biotin) complex, the H3.1–H4 complex and the histone octamer were performed as described previously^58^.

### DNA preparation

DNA for medium- to large-scale individual nucleosome purifications was generated by Phusion (Thermo Fisher Scientific) PCR amplification. The resulting DNA fragment was purified by a Mono Q column (GE Healthcare). All purified DNA was concentrated and stored at −20 °C in 10 mM Tris-HCl pH 7.5 until use. Labelled DNA for smTIRF experiments was also generated using PCR with fluorescently labelled primers (Sigma Aldrich, see Supplementary Table 1).

### Nucleosome assembly

#### Large scale for SeEN-seq, cryo-EM and DNaseI experiments

The DNA and the histone octamer complex were mixed in a 1:1.5 molar ratio in the presence of 2 M KCl. Reconstitution of the H2A–H2B(T122C–biotin)–H3.1–H4 complex was performed by incubating the components at a 1:1.5:3 molar ratio (DNA:H2A–H2B:H3.1–H4). The samples were dialysed against refolding buffer (RB) high (10 mM Tris-HCl pH 7.5, 2 M KCl, 1 mM EDTA and 1 mM DTT). The KCl concentration was gradually reduced from 2 M to 0.25 M using a peristaltic pump with RB low (10 mM Tris-HCl (pH 7.5), 250 mM KCl, 1 mM EDTA and 1 mM DTT) at 4 °C. The reconstituted nucleosomes were incubated at 55 °C (or 37 °C in the case of LIN28-E and Por endogenous nucleosome sequences) for 2 h followed by purification on a Mono Q 5/50 ion-exchange gradient (GE Healthcare), and dialysed into 20 mM Tris-HCl pH 7.5 and 500 μM TCEP overnight. Nucleosomes were concentrated and stored at 4 °C.

#### Small scale for smTIRF experiments

Nucleosomes were prepared following previously established protocols^59^. Typically, 1 µg of labelled biotinylated DNA was combined with recombinant, reconstituted human histone octamers at equimolar ratios in 30 µl TE buffer (10 mM Tris-HCl pH 7.5, 1 mM EDTA) supplemented with 2 M KCl. Then, samples were dialysed overnight from 2 M KCl to 10 mM KCl by Tris-HCl pH 7.5, 1 mM EDTA in dialysis buttons. Samples were collected and centrifuged at 20,000*g* for 10 min at 4 °C and the supernatant was kept on ice. To determine the quality of NCP assemblies, 5% acrylamide native PAGE was run in 0.5× TBE at 90 V on ice for 90 min. Images were taken using ChemiDoc MP (BioRad).

### Protein expression and purification

#### OCT4

Human full-length OCT4 (residues 1–360), was subcloned into pAC-derived vectors^60^ containing an N-terminal Strep II tag. An additional N-terminal EGFP tag and C-terminal sortase-6×His tag (LPETGGHHHHHH) were fused in-frame to improve purification. GFP–OCT4 was expressed in 4-l cultures of *Trichoplusia ni* High Five (Hi5) cells using the Bac-to-Bac system (Thermo Fisher Scientific). Cells were cultured at 27 °C, collected two days after infection, resuspended in lysis buffer (50 mM Tris-HCl pH 8.0, 1 M NaCl, 100 μM phenylmethylsulfonyl fluoride, 1× protease inhibitor cocktail (Sigma) and 250 μM TCEP) and lysed by sonication. The supernatant was collected, and the proteins were purified by Strep-Tactin affinity chromatography (IBA) with a Strep-tag on the N terminus, and then purified by heparin ion-exchange chromatography (GE Healthcare). GFP–OCT4 was further purified by size-exclusion chromatography (Superdex 200; GE Healthcare) in GF buffer (20 mM HEPES pH 7.4, 150 mM NaCl, 5% glycerol, 500 μM TCEP). The purified proteins were concentrated and stored at −80 °C.

#### MYC-MAX bHLH LZ

Both human MYC (UniProtKB P01106, residues 351–437) and human MAX (UniProtKB P61244, 22–102) were subcloned into a pET28-derived vector for co-expression in *Escherichia coli*. MYC contained an N-terminal 6× His tag and MAX remained untagged. Cells were grown aerobically in 4 l LB medium and the respective antibiotics. The cultures were inoculated in a 1:100 (v/v) ratio with an overnight pre-culture and incubated at 37 °C. At an optical density at 600 nm (OD_600 nm_) of 0.6–1, gene expression was induced with 0.5 mM IPTG (final concentration). The cultures were further incubated at 18 °C, 200 rpm overnight, or for 3 h at 37 °C, 200 rpm. Cells were collected by centrifugation at 4 °C for 10 min and stored after shock-freezing in liquid nitrogen at −80 °C. The pellets were resuspended in lysis buffer (50 mM Tris-HCl pH 8, 500 mM NaCl, 3 mM imidazole, 10% (v/v) glycerol and 1× protease inhibitor cocktail (Sigma)) and cells were disrupted by sonification. The supernatant was subjected to a HisTrap HP column (5 ml, GE Healthcare) and then further purified by size-exclusion chromatography (Superdex 200 Increase 10/300 GL; GE Healthcare) in SEC buffer (50 mM HEPES pH 8, 500 mM NaCl, 10% (v/v) glycerol). The purified proteins were concentrated and stored at −80 °C. For smTIRFM experiments, a SpyTag was engineered at the C terminus of MAX and subcloned into the pET28 vector (TWIST Biosciences). Spy-tagged MYC-MAX mutants were generated by site-directed mutagenesis (see Supplementary Table 1), and both wild-type and mutant proteins were purified following the same protocol.

#### MAX-MAX bHLH LZ

Human MAX (residues 2–160) was subcloned into a pET28-derived vector with a Strep II tag for expression in *E. coli*. Protein expression was performed as described for MYC-MAX. The homodimer was res-suspended in 50 mM Tris-HCl pH 8, 500 mM NaCl, 3 mM imidazole, 10% (v/v) glycerol and 1× protease inhibitor cocktail (Sigma)) and cells were disrupted by sonification. The supernatant was subjected to a Strep-Tactin sepharose column (5 ml, GE Healthcare) and then further purified by size-exclusion chromatography (Superdex 200 Increase 10/300 GL; GE Healthcare) in SEC buffer (50 mM HEPES pH 8, 500 mM NaCl and 10% (v/v) glycerol).

#### CLOCK-BMAL1 bHLH PAS-AB

Mouse CLOCK (UniProtKB O087850) bHLH PAS-AB (residues 26–395) and BMAL1 (UniProtKB Q9WTL8) bHLH PAS-AB (residues 62–441) were cloned into separate pFastbac vectors as described previously^30^. In general, 1–2 l of CLOCK-BMAL1 bHLH-PAS-AB-expressing insect cells (*Spodoptera frugiperda* or Hi5) were pelleted and resuspended in His buffer A (20 mM sodium phosphate buffer pH 8, 200 mM NaCl, 15 mM imidazole, 10% (v/v) glycerol, 0.1% (v/v) Triton X-100 and 5 mM β-mercaptoethanol). Cells were lysed by cell disruption and subsequent sonication for 3 min (15 s on, 30 s off). Lysate was clarified by centrifugation at 45,000 rpm for 45 min. Ni-NTA affinity purification was performed on a 5 ml HisTrap FF (GE Healthcare). After 14-column washes in His buffer A, the column was further washed with 6.5% His buffer B (20 mM sodium phosphate buffer pH 7.5, 200 mM NaCl, 300 mM imidazole, 10% (v/v) glycerol and 5 mM β-mercaptoethanol) for 3 column volumes, before being eluted in buffer B over a 10-column volume (CV) gradient. The relevant fractions were pooled and TEV-cleaved at 4 °C for a minimum of 4 h. The complex was then concentrated to 5–10 ml and re-diluted to 50 ml with heparin buffer A (20 mM sodium phosphate buffer pH 7.5, 50 mM NaCl, 2 mM dithiothreitol and 10% (v/v) glycerol) and loaded onto a HiTrap Heparin HP affinity column (GE Healthcare). After washing with 5 CV of the above buffer, the column was washed with a further 3 CV of 25% heparin buffer B (20 mM sodium phosphate buffer pH 7.5, 2 M NaCl, 2 mM dithiothreitol abd and 10% (v/v) glycerol) before eluting with buffer B over an 8-CV gradient. The relevant fractions were purified by Superdex 200 gel filtration chromatography (GE Healthcare) into 20 mM HEPES buffer pH 7.5, 125 mM NaCl, 5% (v/v) glycerol and 2 mM TCEP. CLOCK-BMAL1 mutants were generated by site-directed mutagenesis and purified following the described protocol. For DREX experiments, BMAL1 bHLH-PAS-AB gene block (TWIST Biosciences) was synthesized with a C-terminal SpyTag and cloned into a pAC8 expression vector with a N-terminal His tag and purified in complex with His–CLOCK bHLH PAS-AB as described above.

#### CLOCK-BMAL1 bHLH

Purification of the CLOCK and BMAL1 bHLH construct was performed as reported previously^61^. In brief, mouse BMAL1 bHLH residues 73–135 and mouse CLOCK bHLH residues 29–89 were cloned into pET28-derived vectors (TWIST Biosciences), each with an additional tryptophan engineered at the C terminus to allow for UV detection. The proteins were each expressed and purified separately using a HisTrap HP column (5 ml, GE Healthcare). After affinity purification, the equimolar ratios of CLOCK bHLH and BMAL1 bHLH were mixed and incubated for around one hour on ice. The heterodimer peak was collected after purification using an S75 10/ 300 GL column.

#### BAF

For the expression and purification of human canonical BAF (cBAF), wild-type full-length Dpf2/BAF45d (UNIPROT ID: Q92785) was cloned in the lentiviral transfer plasmid pHR-CMV-TetO2_3C-Twin-Strep_IRES-EmGFP (Addgene plasmid n.113884) and used as a bait for the other endogenous subunits of the complex. A stable cell line was generated by lentiviral transduction of Expi293TM mammalian cells (Thermo Fisher Scientific)^62^ and successfully infected cells—expressing GFP from the same mRNA as the transgene under control of an internal ribosome entry site (IRES)—were enriched by fluorescence-activated cell sorting (FACS). Cells were then scaled up and collected when the cell density reached a value between 6 × 10^6^ cells per ml and 8 × 10^6^ cells per ml. Nuclear extraction was performed on the basis of the previously established protocol for endogenous cBAF purification^33^, with some modifications. First, cell pellets were resuspended in hypotonic buffer (10 mM HEPES pH 8, 10 mM KCl, 1.5 mM MgCl_2_, 1 mM DTT and SIGMAFAST Protease Inhibitor Cocktail) and homogenized. The homogenate was then centrifuged (30 min, 4,000*g*, 4 °C) and the packed nuclear volume (pnv) was determined. The pellet was resuspended in 2 pnv of pre-extraction buffer (20 mM HEPES pH 8, 100 mM KCl, 1.5 mM MgCl_2_, 0.2 mM EDTA, 0.1% NP-40, 1 mM DTT and SIGMAFAST Protease Inhibitor Cocktail) and the suspension was centrifuged (10 min, 4,000*g*, 4 °C). The pellet was then resuspended in 0.5 pnv of low-salt buffer (20 mM HEPES pH 8, 20 mM KCl, 10% glycerol, 1.5 mM MgCl_2_, 0.2 mM EDTA, 1 mM DTT and SIGMAFAST Protease Inhibitor Cocktail), followed by the dropwise addition of 0.5 pnv of high-salt buffer (20 mM HEPES pH 8, 1.2 M KCl, 10% glycerol, 1.5 mM MgCl_2_, 0.2 mM EDTA, 1 mM DTT and SIGMAFAST Protease Inhibitor Cocktail). The solution was incubated for 1 h at 4 °C under rotation, and then centrifuged for 1 h at 25,000 rpm. The supernatant was filtered sequentially trough 1.2-, 0.45- and 0.2-mm filters and loaded on a 5-ml Strep-Tactin XT 4Flow high-capacity column (IBA Lifesciences). The protein was further purified using a 1 ml Mono Q 5/50 GL column (GE Healthcare), followed by a Superose 6 Increase 10/300 GL column (GE Healthcare) and eluted in 20 mM HEPES pH 8, 100 mM KCl, 0.5 mM MgCl_2_, 5% glycerol and 0.5 mM TCEP.

#### cGAS

Truncated human cGAS (155–522) wild-type protein was expressed and purified from *E. coli* strain BL21 (DE3) as decribed previously^34^.

### Labelling of the MYC-MAX variants with the SpyCatcher/SpyTag system

A mutant version of the SpyCatcher protein (SpyCatcherS50C) was purified following previously established protocols^63,64^. SpyCatcherS50C was incubated with DTT (8 mM) at 4 °C for 1 h. DTT was removed using a S200 16/60 gel filtration column (GE healthcare) in a buffer containing 50 mM Tris-HCl pH 7.3 and 150 mM NaCl. JF549-maleimide (Tocris) was dissolved in 100% DMSO and mixed with SpyCatcher to achieve a fourfold molar excess of JF549-maleimide. SpyCatcher was labelled at room temperature for 3 h in a vacuum desiccator and stored overnight at 4 °C. Labelled SpyCatcher was separated from free dye on a S200 16/60 gel filtration column in 50 mM Tris-HCl pH 7.5, 150 mM NaCl, 250 μM TCEP and 10% (v/v) glycerol, concentrated, flash-frozen in liquid nitrogen and stored at −80 °C. Purified wild-type MYC-MAX–Spy, MYC-MAX^Y73A-R76A^–Spy and MYC^S405Y-A408R^-MAX–Spy were mixed with JF549–SpyCatcher in a 5:1 molar ratio and incubated for 1 h at room temperature, frozen in liquid nitrogen.

### smTIRF microscopy experiments

Measurements were performed as described previously^65^. In brief, objective-type smTIRF was performed using a Nikon Ti-E inverted fluorescence microscope, equipped with a CFI Apo TIRF 100× oil immersion objective (NA 1.49), an ANDOR iXon EM-CCD camera and a TIRF illuminator arm. Laser excitation was realized using a Coherent OBIS 640LX laser (640 nm, 40 mW) and coherent OBIS 532LS laser (532 nm, 50 mW). For all smTIRF experiments, flow channels were prepared as described before^65^, washed with 500 µl degassed ultrapure water (Romil), followed by 500 µl 1× T50 (10 mM Tris pH 8, 50 mM NaCl) and background fluorescence was recorded with both 532 nm and 640 nm excitation. Fifty microlitres of 0.2 mg ml^−1^ neutravidin was then injected and incubated for 5 min and washed using 500 µl 1×T50. Then, 50 pM of Alexa647-labelled DNA or NCPs in T50 with 2 mg ml^−1^ bovine serum albumin (BSA, Carlroth) was flowed into the channel for immobilization. Five hundred microlitres of 1× T50 was used to wash out unbound DNA, and 1–2 nM JF549-labelled MYC-MAX was flowed in using imaging buffer (20 mM Tris-HCl pH 7.5, 150 mM NaCl, 10% (v/v) glycerol, 0.005% (v/v) Tween-20, 2 mM Trolox, 3.2% (w/v) glucose, 1× glucose oxidase/catalase oxygen scavenging system and 1 mg ml^−1^ BSA), and movies were recorded at 2–5 Hz in TIRF illumination, alternating between far-red and green illumination (1:200 frames).

### smTIRF microscopy data analysis

Single-molecule trace extraction and trace analysis were done as described previously^65^ with some adjustments. Movies were background-corrected using a rolling ball algorithm in ImageJ. DNA positions were detected using a custom-built MATLAB (Mathworks) script using a local maxima approach. Images were aligned to compensate for stage drift. Fluorescence intensities (in the orange channel) were extracted within a 2-pixel radius of the identified DNA peaks. Individual detections were fitted with a 2D-Gaussian function to determine colocalization with immobilized DNA. Detections exceeding a PSF width of 400 nm, a 250 nm offset from the DNA position or an intensity greater than 5,000 counts were excluded from further analysis. Individual traces were analysed by a step-finding algorithm^66^, followed by thresholding. Overlapping multiple binding events were excluded from the analysis. For each movie, cumulative histograms were constructed from detected bright times (*t*_bright_) corresponding to bound MYC-MAX molecules to obtain dwell times and dark times (*t*_dark_) to obtain on-rate constants, usually including data from around 100 individual traces. The cumulative histograms from traces corresponding to individual DNA were fitted with either di- or tri-exponential functions.

### TR-FRET

LANCE TR-FRET assays were performed with His-tagged MYC-MAX (acceptor, ULight α-6×His antibody) and donor biotinylated nucleosomes (LANCE Eu-W8044 streptavidin) following the general protocol described previously^42^. To analyse His–MYC-MAX binding to the NCP^SHL+5.1^ nucleosomes, biotin was incorporated into H2B (residue T122) using maleimide chemistry (see also the Methods subsection ‘Expression, purification and reconstitution of human octamer histones’). For all other TR-FRET experiments, the biotin was incorporated into the nucleosome using a biotinylated primer proximal to the E-box motif during PCR to produce the DNA fragment (Microsynth). In the MYC-MAX forward titrations, increasing concentrations of His–MYC-MAX (mixed 1:20 with the ULight α-6×His antibody) were added to a mixture of 1 nM biotinylated nucleosome, 2 nM Lance Eu-streptavidin in a buffer containing 20 mM Tris-HCl, pH 7.5, 125 or 75 mM NaCl, 5% glycerol, 0.01% NP-40, 0.01% CHAPS, 5 mM DTT and 100 μg ml^−1^ BSA (T75). Before TR-FRET measurements, reactions were incubated for 5 min at room temperature. For competition experiments with CLOCK-BMAL1, increasing amounts of untagged CLOCK-BMAL1 bHLH PAS-AB wild-type and mutant proteins were incubated with a preformed complex of His–MYC-MAX-nucleosome (625 nM His–MYC-MAX:31.25 nM ULight) in the T75 buffer. After excitation of europium fluorescence at 337 nm, emissions at 620 nm (europium) and 665 nm (ULight) were measured with a 75-μs delay to reduce background fluorescence and the reactions were followed by recording 30 data points of each well over 30 min using a PHERAstar FS microplate reader (BMG Labtech). The TR-FRET signal of each data point was extracted by calculating the 620:665 nm ratio. The signal was corrected for direct acceptor excitation by subtracting the signal observed in the absence of the nucleosome. The resulting raw signals were fitted to the Bmax values of 1 in Prism 7 (GraphPad), assuming equimolar binding of the TF–nucleosome substrates using a one-site specific binding curve.

### Mass photometry

For measuring nucleosomes or nucleosome complexes, microscope coverslips were treated with 10 ul of poly-l-lysine for 30 s, rinsed with Milli-Q and dried under an air stream. Before mass photometry measurements, protein dilutions were made in MP buffer (20 mM Tris-HCl pH 7.5, 100 mM KCl and 0.5 mM TCEP) and nucleosome–TF complexes were mixed in a 1:6 ratio and incubated for 30 min at room temperature. Data were acquired on a Refeyn OneMP mass photometer. First, 18 μl of MP buffer was introduced into the flow chamber and focus was determined. Then 2 μl of protein solution were added to the chamber and movies of 60 or 90 s were recorded. Nucleosomes (NCP^SHL+5.8^, NCP^SHL−6.2^, NCP^POR1^ and NCP^SHL+5.8-tandem^) and CLOCK-BMAL1 bHLH PAS-AB were measured individually at 20 nM (final concentration) and then in complex at 10 and 60 nM, respectively. Each sample was measured at least two times independently (*n* = 2). All acquired movies were processed and molecular masses were analysed using Refeyn Discover 2.3, based on a standard curve created with BSA and thyroglobulin.

### Gel EMSAs

Cy5-labelled nucleosomes (30 nM) were mixed with either CLOCK-BMAL1 bHLH PAS-AB wild type or mutants (0–500 nM), CLOCK-BMAL1 bHLH PAS-AB (250 nM) in the presence and absence of increasing concentrations of cGAS (18.75–150 nM) or cGAS only (75 nM).

For BAF competition assays, unlabelled nucleosomes (30 nM) containing an E-box motif at SHL+5.8 were mixed with BAF only (100 nM), BAF (100 nM) in the presence of increasing amounts of CLOCK-BMAL1 (125 nM, 250 nM and 500 nM) or CLOCK-BMAL1 only (250 nM and 500 nM).The reactions were conducted in binding buffer (BB) (20 mM Tris-HCl pH 7.5, 75 mM NaCl, 10 mM KCl, 1 mM MgCl_2_, 0.1 mg ml^−1^ BSA and 1 mM DTT) and incubated at room temperatute for around one hour. After incubation, the samples were analysed by electrophoresis on a 6% non-denaturing polyacrylamide gel (acrylamide:bis = 37.5:1) in 0.5× TGE buffer (12.5 mM Tris base, 96 mM glycine and 500 μM EDTA), and the bands were visualized with an Odyssey (LiCor) imaging analyser or with a Typhoon FLA 9500 after staining in SYBR GOLD Nucleic Acid Gel Stain (Invitrogen). Fluorescently labelled nucleosomes and DNA-binding curves were analysed using the Empiria Studio v.2.3 software.

### SeEN-seq library pool preparation

DNA sequences were generated by replacing the Widom 601 sequence with the canonical consensus JASPAR E-box motif (GGCACGTGTC, MA0819.1, MA0059.1) at 1-bp intervals across the entire modified W601. The E-box motif present in the original Widom 601 positioning sequence at SHL+5.1 was mutated (see Supplementary Table 1). The W601-E-box variant DNA sequences were flanked by EcoRV sites and adapter sequences and ordered as gene fragments from TWIST Biosciences. The individual gene fragments were suspended, pooled equally and cut with EcoRV-HF (NEB), and DNA fragments (153 bp) were purified from an agarose gel using the QIAquick Gel Extraction kit (Qiagen). The W601-E-box DNA pool was spiked with an excess of W601 DNA (1:30 molar ratio; pool:601). The nucleosome pool was assembled and purified as described above.

### SeEN-seq assay

SeEN-seq was performed as before^25^ with some modifications. For SeEN-seq EMSAs, nucleosomes (100 nM) were incubated with a 62.5 nM final concentration of MYC-MAX bHLH LZ (human MYC residues 351–437, human MAX residues 22–102) or 250 nM of CLOCK-BMAL1 bHLH PAS-AB (mouse CLOCK residues 26–395, mouse BMAL1 residues 62–441) in 20-μl reactions containing 20 mM Tris-HCl pH 7.5, 75 mM NaCl, 10 mM KCl, 1 mM MgCl_2_, 0.1 mg ml^−1^ BSA and 1 mM DTT. To compensate for the loss in DNA-binding affinity in the CLOCK-BMAL1 bHLH construct^61^, CLOCK-BMAL bHLH SeEN-seq was performed with around fivefold higher concentrations (1,250 nM) compared to what was used for the PAS-containing construct. The reactions were incubated at room temperature for around 1 h and loaded onto a 6% non-denaturing polyacrylamide gel (acrylamide:bis = 37.5:1) in 0.5× TGE gel and run for 1 h (150 V, room temperature). Gels were then stained with a SYBR gold nucleic acid stain (around 10 min, Invitrogen). DNA bands corresponding to the size of TF-bound and unbound nucleosome complexes were imaged and excised using a C300 gel doc UV-transilluminator (Azure Biosystems). Gel slices were incubated with acrylamide gel extraction buffer (100 μl, 500 mM ammonium acetate, 10 mM magnesium acetate, 1 mM EDTA and 0.1% SDS) and heated (50 °C, 30 min). H_2_O (50 μl) and the QIAquick Gel Extraction kit QG buffer (450 μl, Qiagen) were added and the samples were heated (50 °C, 30 min). Samples were briefly spun and the supernatant containing DNA fragments were transferred to QIAquick Gel Extraction spin columns. Samples were purified according to the manufacturer’s instructions and eluted in H_2_O (22 μl), and the DNA was quantified by Qubit reagent (Thermo Fisher Scientific). Purified DNA (20 μl, around 2–20 ng DNA) was used for NGS library preparation (NEBNext ChIP–seq, E6240S) with dual indexing (E7600S) and no more than 10 cycles of PCR amplification. Purified sequencing libraries were quantified by Qubit reagent (Thermo Fisher Scientific) and the library size was checked on the bioanalyser platform (Agilent) before sequencing on an Illumina MiSeq or NextSeq platform (300 bp paired-end). Sequencing fragments were mapped to the W601 sequence and E-box-motif-containing variants (153 bp) using the Bioconductor package QuasR with default settings^67^, which internally use Bowtie for read mapping^68^. The number of sequence reads aligned to each construct was quantified by the QuasR function Qcount with every construct represented. SeEN-seq enrichments are calculated by determining the fold change between library-size normalized read counts for each 601-E-box variant in the TF-bound and unbound nucleosome fractions. These fold changes represent a relative affinity difference between all positions. In all replicates we were able to capture every motif position, suggesting that the E-box motif does not markedly affect nucleosome stability.

### XL-MS

The TF and the nucleosomes were mixed in a 1.5:1 ratio in MS sample buffer (50 mM HEPES pH 7.5, 150 mM NaCl and 500 μM TCEP) and incubated at room temperature for around 1 h. In the meantime, an aliquot of disuccinimidyl sulfoxide (DSSO) XL reagent (Thermo Fisher Scientific, A33545) was warmed up to room temperature and diluted to a 100 mM stock concentration in anhydrous DMSO by shaking for 5 min, 400 rpm. After incubation, the sample was transferred to a concentrator (Amicon Ultra, Merck Millipore, 10,000 MWCO), DSSO was added and the cross-linking reaction mix was incubated for 1 h at 10 °C, while shaking at 400 rpm. The excess cross-linker was quenched by adding 1 M Tris pH 6.8 (50 mM final concentration) and incubating for an additional hour at room temperature, 400 rpm. The sample was centrifuged (5 min, 14,000*g*) to remove XL reagent and 400 µl of fresh 8 M urea in 50 mM HEPES, pH 8.5 for denaturing and washing were added. This step was repeated twice. Next, reduction/alkylation buffer (50 mM TCEP, 100 mM 2-chloroacetamide) was added (5 mM and 10 mM final concentration respectively) and the sample was incubated for 30 min while shaking at 400 rpm. It was centrifuged for 5 min at 14,000*g* and 400 µl of fresh 8 M urea was added for denaturing and washing. The sample was centrifuged again for 5 min at 14,000*g*. This step was repeated twice with a final centrifugation step of 15 min instead of 5 min to concentrate the sample to around 30 µl. Lys-C was added (0.2 µg µl^−1^ stock, 1:100 enzyme to protein ratio) and the sample was digested for 1.5 h at room temperature while shaking. The sample was diluted fourfold with 50 mM HEPES, pH 8.5. Then, trypsin (0.2 mg ml^−1^ stock, 1:100 enzyme to protein ratio) was added and the sample was incubated overnight at 37 °C, while shaking at 400 rpm. An additional aliquot of trypsin and acetonitrile to a final concentration of 5% was added the next day and the sample was incubated for another 4 h at 37 °C, while shaking at 400 rpm. The sample was transferred into an Eppendorf tube, TFA was added (1% final concentration) and the sample was briefly sonicated and spun down for 5 min at 20,000 g. The supernatant was desalted using a PreOmics iST-NHS kit and concentrated in a speedvac. Samples were reconstituted with 0.1% TFA in 2% acetonitrile.

Samples were analysed by LC–MS in two ways:

1. The equivalent of around 1 μg peptides per sample was loaded onto a uPAC C18 trapping column, and then separated on a 50-cm uPAC C18 HPLC column (connected to an EASY-Spray source (all Thermo Fisher Scientific, columns formerly from Pharmafluidics)) connected to an Orbitrap Fusion Lumos. The following chromatography method was used: 0.1% formic acid (buffer A), 0.1% formic acid in acetonitrile (buffer B), flow rate 500 nl per min, gradient 240 min in total, (mobile phase compositions in % B): 0–5 min 3–7%, 5–195 min 7–22%, 195–225 min 22–80%, 225–240 min 80%.
2. The equivalent of around 5 μg peptides per sample were loaded onto a Vanquish Neo chromatography system with a two-column set-up. Samples were injected with 1% TFA and 2% acetonitrile in H_2_O onto a trapping column at a constant pressure of 1,000 bar. Peptides were chromatographically separated at a flow rate of 500 nl per min using a 3-h method, with a linear gradient of 2–9% B in 5 min, followed by 9–28% B in 120 min, followed by 28–100% B in 20 min, and finally washing for 15 min at 100% B (buffer A: 0.1% formic acid; buffer B: 0.1 formic acid in 80% acetonitrile) on a 15-cm EASY-Spray Neo C18 HPLC column mounted on an EASY-Spray source connected to an Orbitrap Eclipse mass spectrometer with FAIMS (all Thermo Fisher Scientific). In either case, the mass spectrometer was operated in MS2_MS3 mode, essentially according to a previous report^69^. On the Orbitrap Fusion Lumos mass spectrometer, peptide MS1 precursor ions were measured in the Orbitrap at 120-k resolution. On the Orbitrap Eclipse, three experiments were defined in the MS method, with three different FAIMS compensation voltages, −50, −60 and −75 V, respectively, to increase the chances of more highly charged peptides (that is, cross-linked peptides) being identified.

For each experiment, peptide MS1 precursor ions were measured in the Orbitrap at 60-k resolution. In either case, the MS advanced peak determination (APD) feature was enabled, and those peptides with assigned charge states between 3 and 8 were subjected to CID–MS2 fragmentation (25% CID collision energy), and fragments detected in the Orbitrap at 30-k resolution. Data-dependent HCD-MS3 scans were performed if a unique mass difference (Δ*m*) of 31.9721 Da was found in the CID–MS2 scans with detection in the ion trap (35% HCD collision energy).

MS raw data were analysed in Proteome Discoverer v.2.5 (Thermo Fisher Scientific) using a Sequest^70^ database search for linear peptides, including cross-linker modifications, and an XlinkX^69^ search to identify cross-linked peptides. MS2 fragment ion spectra not indicative of the DSSO cross-link delta mass were searched with the Sequest search engine against a custom protein database containing the expected protein components, as well as a database built of contaminants commonly identified during in-house analyses, from MaxQuant^71^, and cRAP (ftp://ftp.thegpm.org/fasta/cRAP), using the target-decoy search strategy^72^. The following variable cross-linker modifications were considered: DSSO hydrolysed/+176.014 Da (K); DSSO Tris/+279.078 Da (K), DSSO alkene fragment/+54.011 Da (K); DSSO sulfenic acid fragment/+103.993 Da (K), as well as oxidation/+15.995 Da (M). Carbamidomethyl/+57.021 Da (C) was set as a static modification. Trypsin was selected as the cleavage reagent, allowing a maximum of two missed cleavage sites, peptide lengths between 4 or 6 and 150, 10 ppm precursor mass tolerance and 0.02 Da fragment mass tolerance. PSM validation was performed using the Percolator node in PD and a target FDR of 1%.

XlinkX v.2.0 was used to perform a database search against a custom protein database containing the expected complex components to identify DSSO-cross-linked peptides and the following variable modification: DSSO hydrolysed/+176.014 Da (K); oxidation/+15.995 Da (M). Cross-link-to-spectrum matches (CSMs) were accepted above an XlinkX score of 40. Cross-links were grouped by sequences and link positions and exported to xiNET^73^ format to generate cross-link network maps.

Cross-links were mapped to the structure models with an in-house script for PyMOL and the ChimeraX plug-in XMAS^74^. Xwalk was used to calculate solvent accessible surface distances^75^.

Data are available through ProteomeXchange^76^ with the identifier PXD033181.

### Cryo-EM sample preparation

Nucleosomes were mixed with molar excesses of the respective TFs in a volume of around 100 μl and incubated at room temperature for 30 min (molar ratios: 1:3:3, NCP^SHL+5.1^:OCT4:MYC-MAX; 1:1.5, NCP^SHL+5.8^:MYC-MAX; 1:1.5, NCP^SHL+5.8^:CLOCK-BMAL1; 1:3 NCP^SHL–6.2^:CLOCK-BMAL1; 1:3, NCP^SHL+5.1^:MAX-MAX; 1:1.5:3, NCP^LIN28-E^: MYC-MAX:OCT4; 1:3, NCP^Por1^:CLOCK-BMAL1) in a binding buffer containing 20 mM HEPES pH 7.4, 1 mM MgCl_2_, 10 mM KCl and 0.5 mM TCEP. The molar ratio used for each considers the number of TF motifs, with an excess of TF, and the relative affinity of each TF for the nucleosome substrate. The sample was then subjected to cross-linking using the GraFix method^77^. For GraFix cross-linking, the TF–NCP complexes were layered on top of a 10%–30% (w/v) sucrose gradient (20 mM HEPES pH 7.4, 50 mM NaCl, 1 mM MgCl_2_, 10 mM KCl, 0.5 mM TCEP) with an increasing concentration (0–0.34% w/v) of glutaraldehyde (EMS) and subjected to ultracentrifugation (Beckman SW40Ti rotor, 30,000 rpm, 18 h, 4 °C). After centrifugation, 100-μl fractions were collected from the top of the gradient and peak fractions were analysed by native PAGE. The peak fractions were combined and sucrose was removed by dialysis into Grafix buffer (20 mM HEPES pH 7.4, 50 mM NaCl, 1 mM MgCl_2_, 10 mM KCl and 0.5 mM TCEP). The resulting sample was concentrated with an Amicon Ultra 0.5-ml centrifugal filter to around 2–7 μM nucleosomes as determined by measuring the DNA concentration at an absorbance of 260 nm. After concentration, 3.5 μl of sample was applied to Quantifoil holey carbon grids (R 1.2/1.3 200-mesh, Quantifoil Micro Tools). Glow discharging was performed in a Solarus plasma cleaner (Gatan) for 15 s in a H_2_/O_2_ environment. Grids were blotted for 3 s at 4 °C at 100% humidity in a Vitrobot Mark IV (FEI), and then immediately plunged into liquid ethane.

### Cryo-EM data collection

Data were collected automatically with EPU 3.0 (Thermo Fisher Scientific) on a Cs-corrected (CEOS) Titan Krios (Thermo Fisher Scientific) electron microscope operated at 300 kV or on a Glacios (Thermo Fisher Scientific) electron microscope at 200 kV (NCP^SHL+5.1^-MAX-MAX and NCP^Por^-CLOCK-BMAL1 only). For the OCT4–MYC-MAX-bound nucleosome structure, zero-energy-loss micrographs were recorded at a nominal magnification of 130,000× using a Gatan K2 summit direct electron detector (Gatan) in counting mode located after a BioQuantum-LS energy filter (slit width of 20 eV). For the other assemblies the acquisition was performed at a nominal magnification of 75,000–96,000× with a Falcon 4 direct electron detector (Thermo Fisher Scientific). All datasets were recorded with an accumulated total dose of 50 e^–^/Å^2^ and the exposures were fractionated into 50 frames. The targeted defocus values ranged from −0.25 to −2.5 μm.

### Cryo-EM image processing

Real-time evaluation along with acquisition with EPU 3.0 (Thermo Fisher Scientific) was performed with CryoFLARE1.10 (ref. ^78^). Drift correction was performed with the RELION 3 motioncorr implementation^79^, in which a motion-corrected sum of all frames was generated with and without applying a dose-weighting scheme. The CTF was fitted using GCTF 1.06 (ref. ^80^) or the patch CTF implementation in cryoSPARC v.3. Particles were picked using crYOLO (1.8.0)^81^, cisTEM (1.0.0 beta)^82^, AutoPick (implemented in RELION)^83^ or cryoSPARC v.3 blob picker^84^.

All datasets were further processed in RELION 3.0 (ref. ^79^), cryoSPARC v.3 or cryoSPARC v.4 in the case of the NCP^Por^ structure^84^ as indicated in each Extended Data figure including two-dimensional (2D) and 3D classification, 3D refinement, particle polishing and CTF refinement. The resolution values reported for all reconstructions are based on the gold-standard Fourier shell correlation curve (FSC) at 0.143 criterion^83,85^ and all the related FSC curves are corrected for the effects of soft masks using high-resolution noise substitution^86^. The software used for the final refinements of each map is indicated in the corresponding Extended Data figure. For the NCP^SHL–6.2^-CLOCK-BMAL1 map, a composite map of two refinements was generated using combine_focus_maps implementation in PHENIX^87^. LocScale implemented in CCPEM (v.1.5)^88,89^ was used for sharpening and blurring the following maps: NCP^SHL+5.8^-CLOCK-BMAL1, NCP^SHL+5.8^-MYC-MAX and NCP^SHL+5.1^-MYC-MAX-OCT4. The NCP^SHL–6.2^-CLOCK-BMAL1 maps were filtered based on local resolution using cryoSPARC v.3. All local resolutions were estimated with MonoRes (XMIPP) implementation in cryoSPARC v.3 (ref. ^90^).

### Model building and refinement

For modelling of MYC-MAX bound to the NCP in the presence of OCT4, PDB 6T90 (ref. ^25^) was used as a template for the OCT4-bound NCP, and coordinates extracted from PDB 1NKP (ref. ^2^) were used to obtain a template for DNA-bound MYC-MAX. The two models were fitted into the cryo-EM map using ChimeraX (fit-in-map tool; ref. ^56^). The gap between NCP DNA and MYC-MAX DNA was closed using ideal B-form DNA in Coot (v.0.9.6)^91^ and the DNA sequence was adapted accordingly. The joined DNA was refined in PHENIX^92^ using DNA restraints (base pair, stacking). MYC-MAX together with the detached DNA end as well as OCT4 together with the other DNA end were further relaxed into the density using ChimeraX/ISOLDE^93^ in combination with adaptive distance restraints. Side chains were corrected in Coot and ChimeraX/ISOLDE (v.1.2–v.1.5) if necessary. The model coordinates and B-factors were refined using the Rosetta FastRelax and B-factor protocols (v.3.13)^94^ in combination with self-restraints (torsions) and with side-chain repacking disabled. The model for MYC-MAX bound to SHL+5.8 was obtained by docking the NCP template (PDB: 6T93)^25^ into the map and fitting the DNA end with ISOLDE (in combination with adaptive distance restraints). The DNA sequence was adjusted and the MYC-MAX model (PDB: 1NKP; ref. ^58^) was docked by superposition on the E-box motif. The model was further refined with ISOLDE using adaptive distance restraints for different rigid groups (MYC-MAX in combination with released DNA, histones) as well as PHENIX (v.1.19–v.1.20.1) and Rosetta as described above. Putative side-chain density did not allow unambiguous differentiation between MYC-MAX in the quasi-homodimeric overall structure. Therefore, both orientations (MYC-MAX dimer flipped in respect to the nucleosome) were modelled with 50% occupancy, respectively, and side chains were truncated.

In the case of both NCP-bound CLOCK-BMAL1 models, PDB 6T93 (ref. ^25^) was used as the NCP template, PDB 4H10 (ref. ^61^) as the template for the DNA-bound bHLH domains of CLOCK-BMAL1, and PDB 4F3L (ref. ^3^) as the template for the CLOCK-BMAL1 PAS domains. The DNA sequence of the NCP template (6T93) was extended at both ends with ideal B-form DNA generated in Coot and the sequence was adjusted to the construct used in this study. The NCP model was fitted into the cryo-EM density with ChimeraX (fit-in-map tool)^56^ and the detached DNA ends were semi-flexibly fitted into the density with ISOLDE^93^ in combination with adaptive distance restraints. The DNA was refined with PHENIX^92^ and Rosetta^94^ as described for the MYC-MAX structure. The PAS domains from 4F3Lwere docked and rigid-body-refined with phenix.dock_in_map. Again, adaptive distance restraints were generated in ISOLDE for separate groups including the bHLH domains together with the detached DNA segment, the opposite DNA end and the PAS domains. This allowed the groups to be semi-flexibly relaxed into the density while maintaining the original geometry.

In the case of CLOCK-BMAL1 bound to position SHL–6.2, the DNA/bHLH model (4H10) and the NCP template (6T93) were fitted into the density and the DNAs were connected with an ideal B-form DNA generated in Coot. The DNA sequence was adapted to the position SHL–6.2 construct and refined as described for the NCP-bound MYC-MAX structure. The PAS domains from 4F3L were manually docked into the density guided by the cross-link between BMAL1 K212 and H3 K57. Because accurate fitting was not possible owing to local resolution limitations and diffuse map density, the PAS domains were docked against the histones using the Rosetta local docking protocol^95^ in combination with Rosetta density scoring (8°, 3 Å perturbations) and a filter for a maximum cross-link distance of 30 Å between Cα atoms of BMAL1 K212 and H3 K57. The resulting poses were ranked by interface energy and density scores and the pose with the best interface energy score was selected because it was separated from the bulk of other poses while also having a good density score. B-factors were refined as described above. Because of insufficient local resolution, side chains were removed from the CLOCK-BMAL1 models for deposition.

In the case of CLOCK-BMAL1 bound to *Por*, the E-box 1 protomer and the bHLH domain of the E-box 2 protomer were resolved to a resolution facilitating model building. The model from the SHL+5.8 structure was used as a template and readily fit the density of the nucleosome and the internal CLOCK-BMAL1 heterodimer. The DNA sequence was adjusted and the external-bound CLOCK-BMAL1 heterodimer was docked in ChimeraX on the basis of cross-linking data, map fit and orientations of the connecting segments of the PAS domains in respect to the bHLH domains. The model was subjected to semi-flexible fitting with ISOLDE using distance and torsion restraints and further refined with PHENIX using coordinate restraints. Observed inter-CLOCK-BMAL1 cross-links can occur either within a heterodimer or between the heterodimers. Some cross-links would be sterically implausible to occur within the heterodimer and could reflect potential inter-heterodimer cross-links. Together with a histone cross-link (external CLOCK K205 and H3 K56) these putative inter-heterodimer cross-links suggest an overall orientation in which the external CLOCK PAS domains face the internal BMAL1 PAS domains. It was not possible to find a consensus model in which all cross-link distances would be below a threshold of 30 Å. This could be due to the assignment ambiguity of the inter-CLOCK-BMAL1 cross-links or the flexibility of the PAS domains. Because of these ambiguities and the limited local map resolution, the external PAS domains are not included in the final model. B-factors were refined as described above. Because of the insufficient local resolution, side chains were removed from the CLOCK-BMAL1 and histone models for deposition.

The Rosetta cryo-EM refinement protocols were run using an in-house developed pipeline (ROSEM, https://github.com/fmi-basel/RosEM). Validation for all models was carried out with PHENIX^96^ and MolProbity (v.4.5.2)^97^.

### Density map segmentation and figure preparation

Structural figures and cryo-EM segmented maps were produced with UCSF ChimeraX (v.1.3).

### Calculation of clash scores and contact surface area

Clash scores for MYC-MAX–nucleosome and CLOCK-BMAL1–nucleosome models were calculated using a PyMOL script (scanFactor.py) as described previously^45,98^ In brief, a MYC-MAX probe (1NKP) or a CLOCK-BMAL1 probe (4F3L, 4H10) containing an appropriately positioned DNA fragment for superimposing on a nucleosome template model was placed in all possible binding positions, and the clash score for each taken as the total number of atoms in the TF closer than an adjustable threshold distance (1 Å default) to nucleosome atoms.

### DNaseI nucleosome footprinting assay

NCPs reconstituted with Widom 601 DNA containing an E-box motif, at SHL −6.9 and SHL +5.1 and an OCT4 motif at SHL −6.0 were mixed with full-length human OCT4 and/or human MYC-MAX bHLH LZ (human MYC residues 351–437, human MAX residues 22–102) in a 1:2:2 molar ratio in BB buffer (20 mM HEPES pH 7.4, 1 mM MgCl_2_, 10 mM KCl and 0.5 mM TCEP) and incubated on ice for around 30 min. Nucleosomes in the presence or absence of OCT4 and/or MYC-MAX were treated with a titration (0.1 U, 0.5 U) of DNaseI (NEB M0303S) in the presence of MgCl_2_ (2.5 mM) and CaCl_2_ (0.5 mM) for 5 min at 37 °C. The reaction was stopped by adding an equal volume of Stop Buffer (200 mM NaCl, 30 mM EDTA, 1% SDS) and incubated on ice for 10 min. Samples were treated with Proteinase K (10 μg) for 2 h and DNA was retrieved using Ampure Beads (A63881). DNA was used for sequence library preparation (NEBNext ChIP–seq, E6240S) with dual indexing, and sequenced on an Illumina MiSeq (300 bp paired-end). Sequences were mapped to the Widom 601 sequence (147 bp) containing the TF motifs using the Bioconductor package QuasR with default settings^67^, which internally use Bowtie for read mapping^68^. The start position of mapped reads, the DNaseI cut site, was extracted and the counts were binned into 1-bp bins across the length of the W601 sequence. Plots and comparisons were done using 100,000 reads per replicate.

### ChIP

One microgram of genomic DNA extracted from *D. melanogaster* BG-3 cells was assembled into chromatin by adding 15 µl 10× McNAP buffer (0.3 M creatine phosphate, 30 mM ATP, 3 mM MgCl_2_, 1 mM DTT and 10 ng µl^−1^ creatine phosphokinase), 35 µl EX50 buffer (10 mM HEPES/KOH pH 7.6, 50 mM KCI, 1.5 mM MgCl_2_, 50 µM ZnCl, 10% glycerol, 1 mM DTT, 1× Proteinase Inhibitor Complex and 100 µl *Drosophila* preblastoderm embryo extract (DREX, prepared as described previously^37^). Assembly proceeded for 5 h at 26 °C at 300 rpm on a shaking heat block. Then, 250 nM of Spy-tagged proteins were added and allowed to bind for 1 h. Samples were cross-linked with formaldehyde (0.1% final concentration) for 10 min and then quenched by addition of 125 mM glycine. Samples were partially digested by 200 U of micrococcal nuclease (MNase, Sigma) for 2 min. Digestion was stopped by addition of 25 mM EDTA. For immunoprecipitation, samples were precleared on a rotating wheel with 20 µl protein AG beads per 1 µg chromatin for 1 h at 4 °C. Two µl of hIgG1-FcSpyCatcher3 (BioRad TZC009) was added and the reaction was incubated on a rotating wheel at room temperature for 1 h. Then, freshly washed protein AG beads (Helmholtz Centre Munich, monoclonal facility) were added and the incubation continued overnight at 4 °C. The beads were washed 4 times for 5 min with 1 ml of 1× RIPA buffer (1 µg chromatin on 20 µl beads). The beads then were suspended in 100 µl 1× TE buffer and digested with 10 µg RNAse A (Sigma) for 30 min at 37 °C. Then, 100 µg Proteinase K (Qiagen) was added and samples were digested and de-cross-linked overnight at 65 °C while shaking. Beads were pelleted at 1,000*g* for 1 min and the supernatant was transferred to a fresh tube. DNA was purified by two extractions with phenol:chloroform:isoamyl-alcohol (25:24:1, Sigma Aldrich) precipitation and a 70% ethanol wash and dissolved in 10 mM Tris/NaCl, pH 8. Concentrations were determined using Qubit (Thermo Fisher Scientific).

NGS libraries were prepared using the NEBNext Ultra II DNA Library (New England Biolabs) according to the manufacturer’s instructions and sequenced on an Illumina NextSeq1000 sequencer. About 20 million paired-end reads were sequenced per sample for each of the ChIP replicates. Replicates were performed using a separate batch of purified proteins and DREX extracts. Base calling was performed by Illumina’s RTA software, v.1.18.66.3.

### DREX ChIP data analysis

#### Read processing

Sequence reads were demultiplexed by JE demultiplexer^99^ using the barcodes from the Illumina Index read files. Demultiplexed files were aligned to the *D. melanogaster* release 6 reference genome (BDGP6) using Bowtie2 (ref. ^100^) v.2.2.9. (parameter “--end-to-end --very-sensitive --no-unal --no-mixed --no-discordant -X 400”) and filtered for quality using SAMtools 1.6 (ref. ^101^) with a MAPQ score cut-off of -q 2.

#### Replicate correlation

Replicate correlation was determined by first searching the dm6 genome for 5,000 best hits of the CACGTG E-Box motif by FIMO^102^. Then, each replicate was down-sampled to receive the same number of reads per replicate, and reads per motif were counted and plotted against each other. If replicates were sufficiently similar, the sampled reads were merged and used for further analysis. This allowed us to avoid normalization against an input and to retain individual read information.

#### Peak calling

Peaks were called using Homer^103^ v.4.9.1 calling the functions makeTagDirectory (parameters -single -fragLength 150) and findPeaks (parameters -style factor -size 150 -F 6) using the corresponding control samples in which the ChIP was done in the absence of added target TF.

#### De novo motif discovery

Enriched motifs in peak region were discovered using MEME^102^ (v.5.0.2, parameters -mod zoops -dna -revcomp -nmotifs 3). The location of the found motif was used to center the subsequent V-plots to the motif as opposed to the peak centre.

#### V-plots

V-plots were done using the Vplotr library from Bioconductor^104^.In short, the fragment size of each read was plotted relative to the location of the binding motif within each peak. This was done for each sample at its own set of peaks so that only bound sites are shown. Then fragment distributions of all peaks for each sample were merged. Data of MSL2 ChIP–seq were taken from a previous study^38^, which is deposited at the GEO under ascension number GSE169222.

The ‘V’ shape results from the protection of the motif from digestion by the bound TF and is usually symmetrical if motifs on either DNA strand are cumulated or if the motif is palindromic such as the E-box. All reads inside the V include the motif whereas all reads outside do not.

### SMF

Experiments involving mouse tissue collection were approved by the Texas A&M University Institutional Animal Care and Use Committee. Adult male mice were maintained at a constant temperature of 22–23 °C and relative humidity of 50–60%, with a 12-h light:12-h dark cycle. Wild-type (Charles River strain 027) and *Bmal1*^*−/−*^ (BMKO; Jackson Laboratory strain 009100) mice were both in a C57BL/6Crl background and were euthanized in the middle of the day at ZT6 by isoflurane anaesthesia followed by decapitation. Livers were collected, briefly washed in ice-cold 1× PBS, snap-frozen in liquid nitrogen and stored at −80 °C until further use. Nuclei were extracted as described previously^105^. In brief, frozen mouse liver was grained into powder under liquid nitrogen in a mortar and homogenized in 4 ml of ice-cold 1× PBS. Liver homogenate was mixed with 25 ml of ice-cold sucrose homogenate solution (2.2 M sucrose, 10 mM HEPES pH 7.6, 15 mM KCl, 2 mM EDTA, 1 mM PMSF, 0.15 mM spermine, 0.5 mM spermidine and 0.5 mM DTT). After incubation on ice for 10 min, the liver homogenate sucrose solution was carefully poured on the top of a sucrose cushion solution (2.05 M sucrose, 10% glycerol, 10 mM HEPES pH 7.6, 15 mM KCl, 2 mM EDTA, 1 mM PMSF, 0.15 mM spermine, 0.5 spermidine and 0.5 mM DTT) and centrifuged for 45 min at 24,000 rpm (100,000*g*) at 4 °C using a Beckman SW32Ti rotor. Nuclei were resuspended in SMF wash buffer (10 mM Tris pH 7.5, 10 mM NaCl, 2 mM MgCl_2_ and 0.1 mM EDTA) and washed once with the same buffer.

The SMF protocol was adapted from ref. ^106^ and optimized for mouse liver. For each sample, 250,000 nuclei were washed once with M.CviPI wash buffer (50 mM Tris pH 8.5, 50 mM NaCl and 10 mM DTT) and resuspended in 1 mL of 1× M.CviPI reaction buffer (50 mM Tris pH 8.5, 50 mM NaCl, 300 mM sucrose and 10 mM DTT). Then, 18.75 µl of 32 mM SAM and 200 U of M.CviPI (NEB-M0227L; 50 µl) were added, and the reaction was incubated at 37 °C for 7.5 min in a water bath. The reaction was supplemented with 100 U of M.CviPI (25 µl) and 128 µmol of SAM (4 µl) for a second incubation round of 7.5 min at 37 °C. The methylation reaction was stopped by adding 350 µl of SDS-containing buffer (20 mM Tris, 600 mM NaCl and 1% SDS 10 mM EDTA) and 20 µl of Proteinase K (20 mg ml^−1^), and the mixture was incubated overnight at 55 °C. Genomic DNA was isolated by phenol-chloroform purification and isopropanol precipitation, resuspended in 10 mM Tris pH 7.5 and treated with RNAse A at for 1 h at 37 °C. Two micrograms of genomic DNA were used for bisulfite conversion using the Epitect bisulfite conversion kit (QIAGEN 59124). Ten to twelve nanograms of bisulfite-converted DNA were used to amplify a distal enhancer of the gene *Por* (chr. 5:135,674,788–135,675,224; *Mus musculus* mm10 genome version), using the KAPA HiFi Uracil+ kit (Roche) as in ref. ^106^ (forward primer: GGTTTTTTGAGYATAGAATTTTTTTTTT; reverse primer: CCATCTTCTCTCACTTCTRCCCAAT). PCR products were purified with 1.5× SPRI beads, and around 20 ng was used to generate sequencing libraries using the NEBNext Ultra II Kit. Libraries from three biological replicates of wild-type ZT6 and three biological replicates of BMKO ZT6 were pooled together and sequenced with a MiSeq v.2 Nano Reagent kit (paired-end 250 bp).

### SMF analysis

The PairwiseAligner function in the Bio.Align Python package was used for sequence alignment. The matched, mismatched and gapped alignment conditions were given a score of 1.0, −0.2 and −0.5, respectively. The sum of the alignment score at each position divided by the total alignment length was defined as the final alignment score. Sequences in the paired-end fastq files were pre-selected by aligning the first around 25-nt query sequences to both forward and reverse primer sequences. Reads with a primer final alignment score higher than 0.8 were selected, and full-length paired-end query sequences were aligned to bisulfite-converted target sequence (HCH replaced by HTH, GC replaced by GY, and CG replaced by YG, with Y = pyrimidine, and H = not G). Paired-end sequences with a final alignment score higher than 0.7 were selected to reconstitute the full-length enhancer sequence based on the alignment result (in the overlapping region, nucleotides having a higher quality score were used). Next, PCR duplicates were removed, and an equal number of reads were randomly selected in each sample for downstream analysis (*n* = 1,052 reads per sample to match that of the sample with the lowest amount of unique reads). The methylation information at cytosines of all GCH positions (GpC positions that are not followed by a G, to avoid conflicts with endogenous CpG methylation) was extracted, using 0 or 1 to represent unprotected or protected cytosines, respectively. Reads from all six samples were then clustered using the Binary Matrix Decomposition clustering algorithm^107^, and then parsed according to their relative cluster and genotype. Raw data (fastq) reads are available at Mendeley Data: 10.17632/t7xj4rc62t.1.

### Bioluminescence recording

Wild-type mouse *Bmal1* or mutants (Uniprot: Q9WTL8) were cloned into the mammalian lentiviral expression backbone (Addgene plasmid, 73320) with a modification to include a stop codon in-frame with the EGFP to prevent expression of the fusion protein (TWIST Biosciences). Recombinant lentiviral particles were produced in HEK293T cells (ATCC) using Pax2 and pMD2.5 packaging plasmids. The resulting supernatant was used to transduce *Bmal1*^*−/−*^ PER2::LUC fibroblasts as previously^108^. For selection, 1 μg ml^−1^ puromycin was applied for one week with medium changes every 48 h.

Successfully transduced cells were grown to confluence in 12-well dishes in high-glucose (27.8 mM), glutamax-containing DMEM (GIBCO) supplemented with 10% serum (HyClone FetalClone III, Thermo Fisher Scientific) and penicillin–streptomycin. Reconstituted lines also had 0.5 μg ml^−1^ puromycin to maintain selection. Confluent cultures were kept for up to 4 weeks with the medium refreshed every 7–10 days. Before the start of recording, cells were synchronized by the addition of 100 nM dexamethasome for 1 h and then changed to MOPS-buffered ‘air medium’ (bicarbonate-free DMEM, 5 mg ml^−1^ glucose, 0.35 mg ml^−1^ sodium bicarbonate, 0.02 M MOPS, 100 μg ml^−1^ penicillin–streptomycin, 1% Glutamax, 1 mM luciferin, pH 7.4, 325 mOsm (ref. ^109^). Cells were then transferred to an Alligator system (Cairn Research), in which bioluminescent activity was recorded at 15-min intervals using an electron multiplying charge-coupled device (EM-CCD) at constant 37 °C.

Bioluminescent traces of cells were fitted with damped cosine waves using the following equation:

$$y=mx+c+{\rm{Amplitude}}\cdot e-kx\cdot \cos (2\pi (x-{\rm{phase}}){\rm{period}})$$

where *y* is the signal, *m* is the gradient of the detrending line, *c* is the *y* intercept of this detrending line, *x* is the corresponding time, amplitude is the height of the peak of the waveform above the trend line, *k* is the decay constant (such that 1/*k* is the half-life), phase is the shift relative to a cos wave and the period is the time taken for a complete cycle to occur.

### Western blotting

Samples were run on AnyKD Mini-PROTEAN TGX gels (BioRad) using the manufacturer’s protocol with a Tris-Glycine SDS buffer system. Protein transfer to nitrocellulose was performed using the Trans-Blot Turbo Transfer system (BioRad), with a standard or high-molecular weight protocol as appropriate. Nitrocellulose was washed briefly, and then blocked for 30 mins at room temperature in 5% w/w non-fat dried milk (Marvel) in Tris-buffered saline/0.05% Tween-20 (TBST). Membranes were then incubated, rocking, with 1:4,000 primary antibody (M2 anti-Flag, Sigma F3165) to detect CLOCK-BMAL1 and anti-GAPDH (Santa Cruz Biotechnologies sc-365062) was used as a loading control at a dilution of 1:3,000 in blocking buffer (5% milk, TBST) overnight at 4 °C. The following day, the membrane was washed for a further 3 × 10 min in TBST and incubated again for one hour with anti-mouse HRP secondary antibody (Sigma, A9917, 1:5,000). A further 3 × 10-min washes in TBST were performed before chemiluminescence detection using Immobilon reagent (Millipore), which was imaged using a ChemiDoc XRS+ imager (BioRad). Quantification was performed using Image Lab Software 6.0 (BioRad).

### Reporting summary

Further information on research design is available in the Nature Portfolio Reporting Summary linked to this article.

## Online content

Any methods, additional references, Nature Portfolio reporting summaries, source data, extended data, supplementary information, acknowledgements, peer review information; details of author contributions and competing interests; and statements of data and code availability are available at 10.1038/s41586-023-06282-3.

### Supplementary information

Supplementary Information This file contains Supplementary Table 1 (DNA sequences and primers used in this study) and Supplementary Figure 1 (Raw gels).

Reporting Summary

Supplementary Table 2 Cross-linking mass spectrometry data for MYC-MAX and CLOCK-BMAL1–nucleosome complexes.

Supplementary Table 3 Processed DNA sequencing reads for single-molecule footprinting of the *Por* enhancer locus in the mouse liver.

## Figures and Tables

**Extended Data Table 1:**
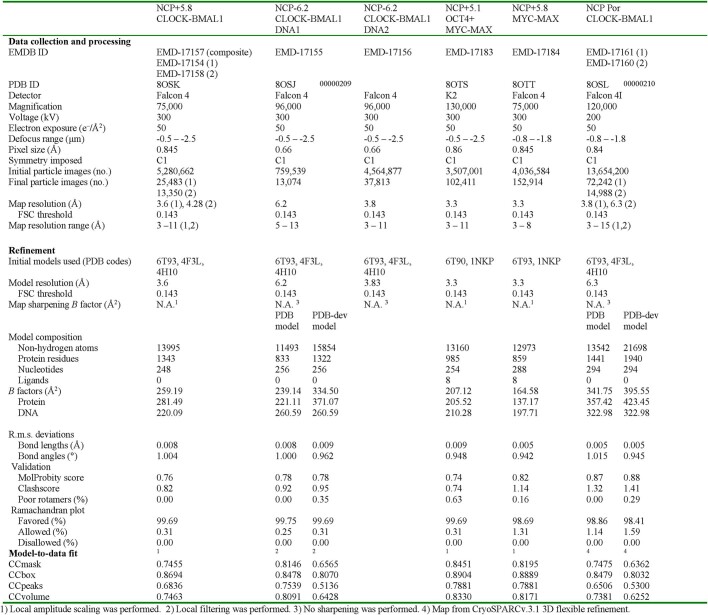
Cryo-EM data collection and refinement statistics

Cryo-EM data collection and refinement statistics of the MYC-MAX–nucleosome and CLOCK-BMAL1–nucleosome complex structures. Coordinate model statistics are only indicated once per deposited structure.

## Data Availability

The electron density reconstructions and final models have been deposited into data banks with the following codes: the Electron Microscopy Data Bank, EMD-17157, EMD-17154, EMD-17158, EMD-17155, EMD-17156, EMD-17183, EMD-17184, EMD-17161 and EMD-17160; the PDB, 8OSK, 8OSJ, 8OTS, 8OTT and 8OSL; and PDB-Dev, PDBDEV_00000209 and PDBDEV_00000210. ChIP–seq data of MYC-MAX and CLOCK-BMAL1 on in vitro reconstituted chromatin have been deposited with the GEO accession code GSE224589. Raw data sequencing reads for the SMF analysis have been deposited to Mendeley Data: 10.17632/t7xj4rc62t.1. We used previously published, and public, sequencing datasets (GSE39860) for the BMAL1 mouse ChIP–seq analysis. XL-MS data are available through ProteomeXchange with identifier PXD033181.

## Code availability

Custom code for DREX experiments can be accessed at https://github.com/nikolas848/eggers_2023_nature. See https://github.com/aliciamichael/amichael/blob/master/scanFactor_var_super.py for the TF-clash analysis and https://github.com/fmi-basel/RosEM for Rosetta cryo-EM refinement protocols. The script used for SMF analysis has been deposited at the Mendeley Data repository: 10.17632/t7xj4rc62t.1.
