# Supplementary material for: Cooperation between bHLH transcription factors and histones for DNA access
Source: Nature. 2023 Jul 5;619(7969):385–93. doi: 10.1038/s41586-023-06282-3 (PMC10338342; doi:10.1038/s41586-023-06282-3)

nature portfolio

https://doi.org/10.1038/s41586-023-06282-3

Supplementary information

Cooperation between bHLH transcription factors and histones for DNA access

In the format provided by the authors and unedited

Supplementary Information

Supplementary Table 1. DNA sequences and primers used in this study.

Name	Sequence	Purpose
LIN28-E	ATCAACATATCTTTGTTATGCAAATTTAACATGGAACTTACTCCAACAATACAGATGCTGAATAAATGTAGTCTAAGTGAAGGAAGAAGGAAAGGTGGGAGCTGCCATCACTCAGAATTGTCCAGCACGTGTTGTGCAAGCTTGTGAATAGAT	CryoEM, DnaseI
2XMYC+OCT4	ATCCACGTGGCTTTGTTATGCAAATCGGGGTGGGGCGTCGTAGACAGCTCTAGCACCGCTTAAACGCACGTACGCGCTGTCCCCCGCGTTTTAACCGCCAAGGGGATTACTCCCTAGTCTCCAGGCACGTGTCAGATATATACATCCTGTGAT	CryoEM, DnaseI
W601 E-box mutant	ATCCTGGAGAATCCCGGTCTGCAGGCCGCTCAATTGGTCGTAGACAGCTCTAGCACCGCTTAAACGCACGTACGCGCTGTCCCCCGCGTTTTAACCGCCAAGGGGATTACTCCCTAGTCTCCAGGTCATAATCAGATATATACATCCTGTGAT	TIRF
CLOCK-BMAL1 or MYC-MAX (SHL+5.8)	ATCCTGGAGAATCCCGGTCTGCAGGCCGCTCAATTGGTCGTAGACAGCTCTAGCACCGCTTAAACGCACGTACGCGCTGTCCCCCGCGTTTTAACCGCCAAGGGGATTACTCCCTAGTCTCCAGGCACGGGTCACGTGCATACATCCTGTGAT	CryoEM
CLOCK-BMAL1 (SHL-6.2)	ATCCTGGAGGGTCACGTGCTGCAGGCCGCTCAATTGGTCGTAGACAGCTCTAGCACCGCTTAAACGCACGTACGCGCTGTCCCCCGCGTTTTAACCGCCAAGGGGATTACTCCCTAGTCTCCAGGCACGTGTCAGATATATACATCCTGTGAT	CryoEM
Alexa647-1XMYC (NCP$^{SHL+5.1}$)	Alexa647-GGGATCCTAATGACCAAGGAAAGCATGAATTCTTCACACCCTGGAGACTTTGTTATGCAAATCCGCTCAATTGGTCGTAGACAGCTCTAGCACCGCTTAAACGCACGTACGCGCTGTCCCCCGCGTTTTAACCGCCAAGGGGATTACTCCCTAGTCTCCAGGCACGTGTCAGATATATACATCCTGTGAGTTCATCCCTTATGTGATGGTACCCTATACGCGGCCGC-Biotin	smTIRFM
Alexa647-E-box_mutant-biotin	Alexa-647-GGGATCCTAATGACCAAGGAAAGCATGAATTCTTCACACCCTGGAGACTTTGTTATGCAAATCCGCTCAATTGGTCGTAGACAGCTCTAGCACCGCTTAAACGCACGTACGCGCTGTCCCCCGCGTTTTAACCGCCAAGGGGATTACTCCCTAGTCTCCAGGTCATAATCAGATATATACATCCTGTGAGTTCATCCCTTATGTGATGGTACCCTATACGCGGCCGC-Biotin	smTIRFM
R173A forward	TGACGCAGGGAAGATCCTCTTTGTCTC	SDM Bmal1
R173A reverse	CATCCTACGACAAACAAAAATCCATCT	SDM Bmal1

K212A forward	AGTTGCGGAACAGCTATCTTCCTCGGACAC	SDM Bmal1
K212A reverse	TTGGCAATATCTTTTGGATGCAGGTAGTC	SDM Bmal1
Q385A forward	TCATGCAGACGACATAGGACACCTCGCA	SDM Bmal1
601 forward	ATCCTGGAGAATCCCGGTCTGCAGG	Large scale DNA
601 reverse	ATCACAGGATGTATATATCTGACACGTGCCT	Large scale DNA
2XMYC+OCT4 forward	ATCCACGTGGCTTTGTTATGCAAATCG	Large scale DNA
1XCLOCK-BMAL1 or MYC-MAX (SHL+5.8) reverse	ATCACAGGATGTATGCACGTGACCCGTGCCT GGAGACTAGG	Large scale DNA
CLOCK-BMAL1 (SHL-6.2)	ATCCTGGAGGGTCACGTGCTGCAGGCCGCTC AA	Large scale DNA
Labelled 1XMYC (SHL+5.1) forward	[Atto647N]-GGGATCCTAATGACCAAGGAAAGCATGAA	Large scale DNA
Labelled 1XMYC (SHL+5.1) reverse	[Biotin]-GCGGCCGCGTATAGGGTACCAT	Large scale DNA
*myc-max*Y73A R76A forward	GACAAA**GCA**ACCGAGTACATCCAG**GCT**ATGC GT	MAX SDM
*myc-max*Y73A_R76A reverse	ATGAGT**GTG**GTTCTT**AGC**ACGCATAGCCTGG AT	MAX SDM
*myc*S405Y A408R *max* forward	GAAAGCCACTGCATACATTCTG**TAC**GTGCAG **CGC**GAA	MYC SDM
*myc*S405Y A408R *max* reverse	CTCGCTGATCAGTTTCTGTTCTTC**GCG**CTGCA CGTA	MYC SDM

Supplementary Table 2. Crosslinking mass-spectrometry data for MYC-MAX and CLOCK-BMAL1-nucleosome complexes.

Supplementary Table 3. Processed DNA sequencing reads for single-molecule footprinting of the Por enhancer locus in the mouse liver.

Supplementary Figure 1. Raw data (gels).

Extended Data Figure 4f

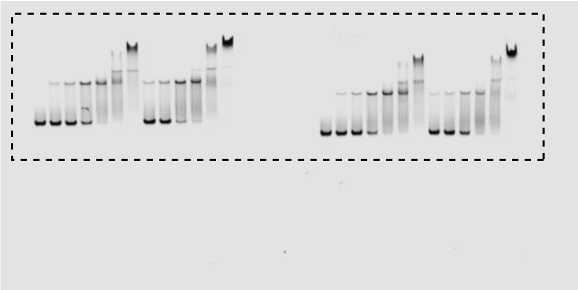

Extended Data Figure 4g

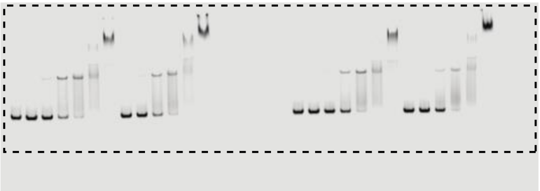

Extended Data Figure 9q

anti-FLAG

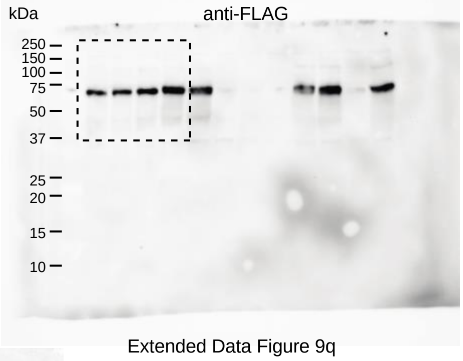

Extended Data Figure 4h

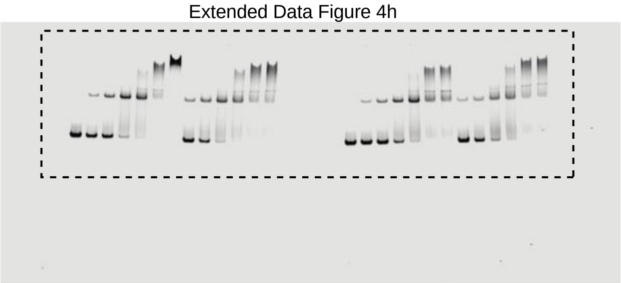

Extended Data Figure 9r

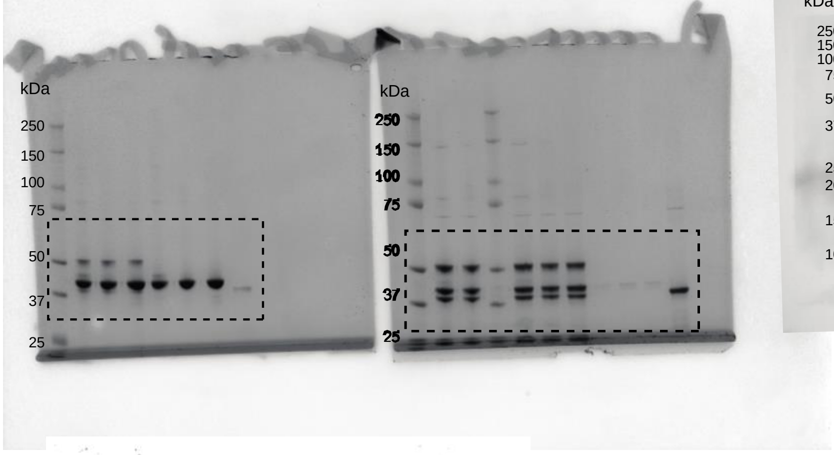

Extended Data Figure 9q

anti-GAPDH

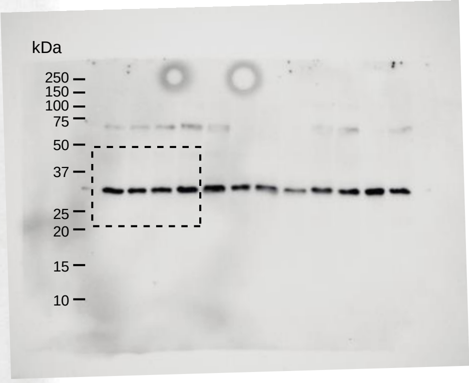

Extended Data Figure 3k

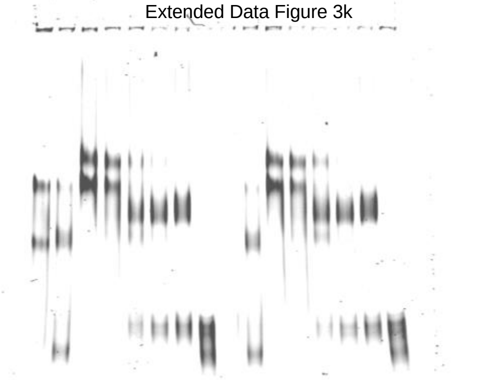

Extended Data Figure 3m

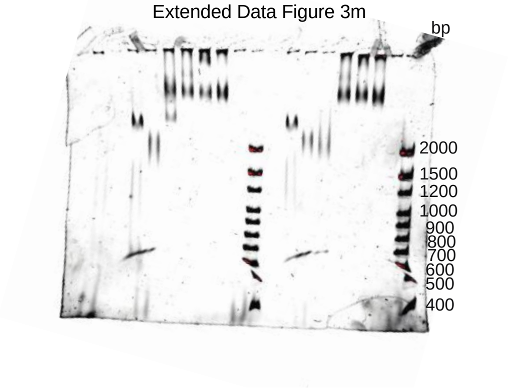

Supplement: Supplementary file 1 — This file contains Supplementary Table 1 (DNA sequences and primers used in this study) and Supplementary Figure 1 (Raw gels). [file 41586_2023_6282_MOESM1_ESM.pdf]
