# Supplementary material for: Cooperation between bHLH transcription factors and histones for DNA access
Source: Nature. 2023 Jul 5;619(7969):385–93. doi: 10.1038/s41586-023-06282-3 (PMC10338342; doi:10.1038/s41586-023-06282-3)
Supplement: Supplementary file 2 — Reporting Summary [file 41586_2023_6282_MOESM2_ESM.pdf]

nature portfolio

Corresponding author(s): Nicolas Thoma

Last updated by author(s): 2/6/2023

Reporting Summary

Nature Portfolio wishes to improve the reproducibility of the work that we publish. This form provides structure for consistency and transparency in reporting. For further information on Nature Portfolio policies, see our Editorial Policies and the Editorial Policy Checklist.

Statistics

For all statistical analyses, confirm that the following items are present in the figure legend, table legend, main text, or Methods section.

n/a	Confirmed	
☐	☒	The exact sample size (*n*) for each experimental group/condition, given as a discrete number and unit of measurement
☐	☒	A statement on whether measurements were taken from distinct samples or whether the same sample was measured repeatedly
☐	☒	The statistical test(s) used AND whether they are one- or two-sided<br>*Only common tests should be described solely by name; describe more complex techniques in the Methods section.*
☐	☒	A description of all covariates tested
☐	☒	A description of any assumptions or corrections, such as tests of normality and adjustment for multiple comparisons
☐	☒	A full description of the statistical parameters including central tendency (e.g. means) or other basic estimates (e.g. regression coefficient) AND variation (e.g. standard deviation) or associated estimates of uncertainty (e.g. confidence intervals)
☐	☒	For null hypothesis testing, the test statistic (e.g. *F*, *t*, *r*) with confidence intervals, effect sizes, degrees of freedom and *P* value noted<br>*Give P values as exact values whenever suitable.*
☒	☐	For Bayesian analysis, information on the choice of priors and Markov chain Monte Carlo settings
☒	☐	For hierarchical and complex designs, identification of the appropriate level for tests and full reporting of outcomes
☐	☒	Estimates of effect sizes (e.g. Cohen's *d*, Pearson's *r*), indicating how they were calculated

Our web collection on statistics for biologists contains articles on many of the points above.

Software and code

Policy information about availability of computer code

Data collection	Illumina RTA 2.4.1 (NextSeq 500 and MiSeq) and bcl2fastq2 v2.17 was used for basecalling and demultiplexing. Cryo-EM datasets were collected using EPU 3.0 (Thermo Fisher)
Data analysis	Data analysis was performed using R 3.6.1 and R/Bioconductor packages: TxDb.Mmusculus.UCSC.mm10.knownGene 3.4.7, Biostrings 2.52.0, QuasR 1.18.0, Rbowtie 1.18.0, TSA 1.2. Reads were mapped by Bowtie (SeEN-seq).<br><br>EM processing was done using CryoFLARE 1.10 (www.cryoflare.org), RELION3.0, Gctf1.06, Motioncorr (Relion implementation), crYOLO (1.8.0), cryoSPARCv3, cryoSPARCv4, cisTEM (1.0.0 beta), AutoPick and cryoSPARCv3 blob picker. All local resolutions were estimated with MonoRes (XMIPP) implementation in cryoSPARCv3. Model building was done using PHENIX (v1.19 – v1.20.1) , RosettaFastRelax (v3.13) was run with an in-house developed pipeline: https://github.com/fmi-basel/RosEM, ISOLDE (v1.2 - v1.5) , and COOT (v0.9.6). LocScale implemented in CCPEM (v1.5) was used for sharpening and blurring. Validation for all models was carried out with MOLPROBITY (v4.5.2). Structural figures were produced with UCSF ChimeraX version 1.3. For transcription factor-nucleosome clash scores a variation of Pymol code is also deposited here: https://github.com/aliciamichael/amichael/blob/master/scanFactor_var_super.py.<br><br>Circadian analysis was performed with eJTK_cycle on the BioDare2 platform: biodare2.ed.ac.uk<br><br>All mass photometry data were analyzed with the Refeyn Discover 2.3 software.<br><br>MS raw data were analyzed in Proteome Discoverer version 2.5 (Thermo Fisher Scientific) using a Sequest database search for linear peptides, including crosslinker-modifications, and an XlinkX v2.0 search to identify cross-linked peptides. MS2 fragment ion spectra not indicative of the DSSO crosslink delta mass were searched with the Sequest search engine against a custom protein database containing the expected protein

components, as well as a database built of contaminants commonly identified during in-house analyses, from MaxQuant 20, and cRAP (ftp://ftp.thegpm.org/fasta/cRAP), using the target-decoy search strategy. Crosslinks were mapped to the structure models with an in-house script for PyMOL and the ChimeraX plugin XMAS (https://doi.org/10.1101/2022.04.21.489026). Xwalk was used to calculate solvent accessible surface distances (http://dx.doi.org/10.1093/bioinformatics/btr348).

For SMTIRM: Movies were background-corrected using a rolling ball algorithm in ImageJ2. DNA positions were detected using a custom-built Matlab (Mathworks) script employing a local maxima approach. Sequential images were aligned using the far-red channel to compensate for stage drift. Fluorescence intensities (in the orange channel) were extracted from the stack within a 2 pixel radius of the identified DNA peaks. Every detected spot in the orange channel was fitted with a 2D-Gaussian function to determine colocalization with immobilized DNA/nucleosome molecules. Residence times were determined using a semi-automatic procedure using Mathlab script. Individual binding events were detected using a thresholding algorithm. For each movie cumulative histograms were constructed from bright times corresponding to bound MYC-MAX molecules in OriginPro2019. The cumulative histograms from traces corresponding to individual DNA/nucleosome molecules were fitted with bi-exponential function, yielding two residence times toff,1 and toff,2. The Mathlab script can be available upon the request from the authors.

For Chip-Seq analysis: Sequence reads were demultiplexed by JE demultiplexer using the barcodes from the Illumina Index read files. Demultiplexed files were aligned to the D. melanogaster release 6 reference genome (BDGP6) using Bowtie2 51 version 2.2.9. (parameter "--end-to-end --very-sensitive --no-unal --no-mixed --no-discordant -X 400") and filtered for quality using samtools 1.6 52 with a MAPQ score cutoff of -q 2. Peaks were called using Homer version 4.9.1. Enriched motifs in peak region were discovered using MEME (version 5.0.2 parameters -mod zoops -dna -revcomp -nmotifs 3). V-plots were done using the Vplotr library from Bioconductor.

For SMF: The pairwiseAligner function in the Align python package was used for sequence alignment. The matched, mismatched, and gapped alignment condition was given a score of 1.0, -0.2 and -0.5, respectively. Reads from all six sample were clustered using the Binary Matrix Decomposition clustering algorithm

EMSA quantification: Empiria Studio v 2.3 software (Li-cor)

Analysis of TR-FRET binding curves was performed in Prism 7 (GraphPad).

For manuscripts utilizing custom algorithms or software that are central to the research but not yet described in published literature, software must be made available to editors and reviewers. We strongly encourage code deposition in a community repository (e.g. GitHub). See the Nature Portfolio guidelines for submitting code & software for further information.

Data

Policy information about availability of data

All manuscripts must include a data availability statement. This statement should provide the following information, where applicable:

- Accession codes, unique identifiers, or web links for publicly available datasets
- A description of any restrictions on data availability
- For clinical datasets or third party data, please ensure that the statement adheres to our policy

Crosslinking data are available via ProteomeXchange with identifier PXD033181.
Models: 8OSK, 8OSJ, 8OTS, 8OTT, 8OSL
cryo-EM maps: EMD-17157, EMDB-17154, EMD-17158, EMD-17155, EMD-17156, EMD-17183, EMD-17184, EMD-17161, EMD-17160
For the SMF experiments: processed sequences (as csv files) are provided as Supplementary Table 3 and raw fastq files have been deposited to Mendeley Data: DOI: 10.17632/t7xj4rc62t.1

Human research participants

Policy information about studies involving human research participants and Sex and Gender in Research.

Reporting on sex and gender	n/a
Population characteristics	n/a
Recruitment	n/a
Ethics oversight	n/a

Note that full information on the approval of the study protocol must also be provided in the manuscript.

Field-specific reporting

Please select the one below that is the best fit for your research. If you are not sure, read the appropriate sections before making your selection.

☒ Life sciences ☐ Behavioural & social sciences ☐ Ecological, evolutionary & environmental sciences

For a reference copy of the document with all sections, see nature.com/documents/nr-reporting-summary-flat.pdf

Life sciences study design

All studies must disclose on these points even when the disclosure is negative.

Sample size	For cryoEM: Sample sizes were not predetermined. The sample size including number of micrographs and separate data collections are described in the EXD figures for each cryoEM map. Micrographs (images) were collected until a sufficient resolution was reached, suggesting sufficient sample size.<br>For biochemical and biophysical studies a sample size determination was not necessary or predetermined.<br>In vitro reconstitution/ChIP-seq assay: No sample size calculation was performed. This is not applicable as we tested purified proteins in biochemical assays. For single-molecule footprinting (SMF) in mouse liver: No statistical methods were used to predetermine sample sizes. We established that our sample size of n = 3 per group are sufficient based on commonly used sample sizes in this field of research, the ability to conduct statistical analyses, and the reproducible SMF patterns between biological replicates.<br>Circadian assay: A minimum sample size of 3 independent cell dishes per bioluminscence recording was used to allow for appropriate statistical calculation within each recording, with 2-4 independent recording experiments performed.<br>SMTIRF: one movie usually included data from 100-200 individual traces.
Data exclusions	For SeEN-seq: all replicates are included in the analysis. For cryo-EM: Through 2D and 3D classification during cryo-EM processing, broken particles or particles that do not belong to the class of interests have been discarded, a standard practice in cryoEM studies to obtain homogeneous high resolution cryoEM structures.<br>In vitro reconstitution/ChIP-seq assay: No data was excluded. For SMF: No data were excluded from the analysis. For SMF clustering analysis, the number of reconstituted full-length reads for some samples was down-sampled (random selection) to match that of library with the lowest number of reads, such that clustering analysis would be performed between six biological samples of equal size, i.e., each sample had an equal weight in the clustering analysis.<br>Circadian assay: No data was excluded.<br>SMTIRF: Peaks exceeding an experimentally determined PSF width for a single JF-549 molecule were excluded from further analysis. Fluorescent traces without any binding (due to the photobleaching/unlabeled protein) were excluded from the analysis.
Replication	CryoEM datasets were collected with multiple samples in separate imaging sessions. Biochemical experiments were repeated at least in three independent experiments and are all reproducible. SeEN-seq enrichment profiles are highly reproducible between technical replicates at single TF concentrations (n=3 replicates, Pearson correlation >0.8). Cellular experiments were also repeated in at least three independent experiments and are all reproducible. We include data for the replicates in our analysis within the main figures and Extended Data files.<br>In vitro reconstitution/ChIP-seq assay: Samples were replicated and correlated well. Values are included in the supplementary information of the manuscript. For SMF: Findings in this manuscript have been replicated in all animals.<br>Circadian assay: Cell lines produced by Bmal1 viral reconstitution were made from at least two independent viral stocks, with 2-4 independent cell lines generated per genotype. Bioluminescent recording was performed a minimum of two times per cell line from independently seeded dishes. All reported results replicated between recordings.<br>SMTIRF: Number of independent replicates (movies) for SMTIRF DNA measurements was 3 for MYC-MAX Y73A,R76A, 4 for MYC S405Y,A408R-MAX, and 7 for MYC-MAX WT, for nucleosome measurements : 4 for MYC-MAX Y73A,R76A and MYC S405Y,A408R-MAX and 7 for MYC-MAX WT.
Randomization	In vitro reconstitution/ChIP-seq assay: Samples were not randomized, this is not applicable. For SMF: Allocation of animals from the same genotype for sample collection was random. Covariates were not relevant in this study because experiments were performed in parallel, and animals were maintained under identical conditions.<br>Circadian assay: There was no group allocation here as genotypes define the group, so randomization is not required.
Blinding	In vitro reconstitution/ChIP-seq assay: Investigators were not blinded, this is not applicable. For SMF: Investigators were not blinded to group allocation during data collection. SMF analysis was fully automated from mapping sequencing reads to the clustering analysis, and blinding was thus not relevant for this study.<br>Circadian assay:There was no group allocation, so this was not blinded. Analysis of bioluminescence traces was performed blind.

Reporting for specific materials, systems and methods

We require information from authors about some types of materials, experimental systems and methods used in many studies. Here, indicate whether each material, system or method listed is relevant to your study. If you are not sure if a list item applies to your research, read the appropriate section before selecting a response.

Materials & experimental systems

n/a	Involved in the study	
☐	☒	Antibodies
☐	☒	Eukaryotic cell lines
☒	☐	Palaeontology and archaeology
☐	☒	Animals and other organisms
☒	☐	Clinical data
☒	☐	Dual use research of concern

Methods

n/a	Involved in the study	
☐	☒	ChIP-seq
☒	☐	Flow cytometry
☒	☐	MRI-based neuroimaging

Antibodies

Antibodies used	FLAG antibody: Sigma (F3165), GAPDH: Santa Cruz antibody (sc-365062).<br>hIgG1-FcSpyCatcher3 (Biorad #TZC009)<br>anti-mouse HRP secondary antibody: (Sigma, A9044)
Validation	Flag antibody. from Manufacturer's website: The monoclonal antibody detects only the target protein band(s) on a Western blot from an E. coli, plant or mammalian crude cell lysate. GAPDH, From manufacturer's website: GAPDH (G-9) is a mouse monoclonal antibody raised against amino acids 1-335 representing full length GAPDH of human origin and fluorescent western blots of crude HeLa lysate and immunohistochemistry of formalin-fixed SW480 was performed. hIgG1-FcSpyCatcher3 from Biorad manufacturer's infromation: antibodies have been validated with knockouts, siRNA and immunoprecipitation followed by mass-spectrometry. HRP antibody: Validated by western blot using HeLa cell lysate according to manufacturer's specifications (Merck).

Eukaryotic cell lines

Policy information about cell lines and Sex and Gender in Research

Cell line source(s)	SSf9 (Thermo Fisher, catalog number: 11496015), Hi5 cells (Thermo Fisher, catalog number: B85502) and HEK 293T (ATCC, CRL-3216) were used in this study. Parental mouse fibroblasts were derived from Bmal1 -/- Per2Luc reporter mice as described in Xu et al. NSMB (2015) All other recombinant cell lines were generated in house for this study.
Authentication	Cell lines were verified by examination of cell morphology under microscope, genotyping by PCR and Western blot, and circadian rhythm phenotyping. HEK293T cells were obtained verified from ATCC. All mammalian cell lines were used within 25 passages of isolation from either the mouse or receipt from ATCC.
Mycoplasma contamination	All parental and recombinant cell lines expressing different genes were tested negative using a mycoplasma Detection Kit (InvioGen). Additionally, mouse fibroblasts were maintained with mycozap to prevent mycoplasma contamination.
Commonly misidentified lines<br>(See ICLAC register)	None of commonly misidentified lines were used in this study.

Animals and other research organisms

Policy information about studies involving animals; ARRIVE guidelines recommended for reporting animal research, and Sex and Gender in Research

Laboratory animals	Adult male wild type (WT) and Bmal1-/- (BMKO) mice, both in C57BL/6Crl strain. Only male mice were used for the SMF analysis.
Wild animals	No wild animals were used in the study.
Reporting on sex	Only male mice were used, but findings hold true for both gender
Field-collected samples	No field-collected samples were used in the study.
Ethics oversight	Experiments involving mouse tissue collection were approved by the Texas A&M University Institutional Animal Care and Use Committee (IACUC AUP #2022-0050). Mice were maintained at a constant temperature of 22-23ºC and relative humidity of 50-60%, and with a 12-hr light : 12-hr dark cycle. Wild type (WT; Charles River strain 027) and Bmal1-/- (BMKO; Jackson laboratory strain 009100) mice were both in C57BL/6Crl background. Food and water was provided ad libitum. Mice were 4-6 month-old at the time of tissue collection.

Note that full information on the approval of the study protocol must also be provided in the manuscript.

ChIP-seq

Data deposition

☒ Confirm that both raw and final processed data have been deposited in a public database such as GEO.

☒ Confirm that you have deposited or provided access to graph files (e.g. BED files) for the called peaks.

Data access links<br>*May remain private before publication.*	We used previously published, and public, sequencing datasets (GSE39860) for the ChIP-Seq analysis. Raw sequencing reads (fastq files), visualization files (bw files), and bed files are all publicly available at https://www.ncbi.nlm.nih.gov/geo/query/acc.cgi?acc=GSE39860.<br>The information below in the methodology section was directly taken from the link provided above.<br>To review GEO accession GSE224589:<br>Go to https://www.ncbi.nlm.nih.gov/geo/query/acc.cgi?acc=GSE224589<br>Enter token kvyhkiomftcnhwv into the box<br><br>For SMF analysis: processed sequences (as csv files) are provided as Supplementary Table 3 and raw fastq files have been deposited to Mendeley Data: DOI: 10.17632/t7xj4rc62t.1

Files in database submission	https://www.ncbi.nlm.nih.gov/geo/download/?acc=GSM982688&format=file&file=GSM982688%5FR1Bmal1%5FBC1%2Ebw<br>https://www.ncbi.nlm.nih.gov/geo/download/?acc=GSM982689&format=file&file=GSM982689%5FR1Bmal1%5FBC2%2Ebw<br>https://www.ncbi.nlm.nih.gov/geo/download/?acc=GSM982690&format=file&file=GSM982690%5FR1Bmal1%5FBC3%2Ebw<br>https://www.ncbi.nlm.nih.gov/geo/download/?acc=GSM982691&format=file&file=GSM982691%5FR1Bmal1%5FBC4%2Ebw<br>https://www.ncbi.nlm.nih.gov/geo/download/?acc=GSM982692&format=file&file=GSM982692%5FR1Bmal1%5FBC5%2Ebw<br>https://www.ncbi.nlm.nih.gov/geo/download/?acc=GSM982693&format=file&file=GSM982693%5FR1Bmal1%5FBC6%2Ebw<br>https://www.ncbi.nlm.nih.gov/geo/download/?acc=GSM982694&format=file&file=GSM982694%5FR1Bmal1%5FBC7%2Ebw<br>https://www.ncbi.nlm.nih.gov/geo/download/?acc=GSM982695&format=file&file=GSM982695%5FR1Bmal1%5FBC14%2Ebw<br>172_chip_neg_1.txt.gz<br>172_chip_neg_2.txt.gz<br>180_chip_neg_1.txt.gz<br>180_chip_neg_2.txt.gz<br>181_chip_neg_1.txt.gz<br>181_chip_neg_2.txt.gz<br>168_chip_mycmax_1.txt.merged.chip_neg.bed<br>merged.chip_mycmax.bed<br>merged.chip_clock.bed<br>chip_clock.ipnorm.bw<br>chip_clock.peaks.bed<br>chip_mycmax.ipnorm.bw<br>chip_mycmax.peaks.bedgz<br>168_chip_mycmax_2.txt.gz<br>181_chip_mycmax_1.txt.gz<br>181_chip_mycmax_2.txt.gz<br>180_chip_clock_1.txt.gz<br>180_chip_clock_2.txt.gz<br>181_chip_clock_1.txt.gz<br>181_chip_clock_2.txt.gz
Genome browser session (e.g. UCSC)	no longer applicable

Methodology

Replicates	3 (SMF)<br>in vitro reconstitution:<br>replicates were generated using a different protein preparation and a different chromatin assemblies on different days. For the IP experiments 2 replicates and for the negative control 3 replicates were summarized.
Sequencing depth	~40,000,000 per samples<br>Samples were sequenced for 15 million 50bp PE reads each using a Next-Seq1000. we received the following number of uniquely mapped reads:<br>8723167 180_chip_clock.bed<br>11724457 181_chip_clock.bed<br>15672876 168_chip_mycmax.bed<br>16312014 181_chip_mycmax.bed<br>9535605 172_chip_neg.bed<br>7358131 180_chip_neg.bed<br>12445404 181_chip_neg.bed
Antibodies	BMAL1 antibody, with chip antibody reference: Lee et al. 2001, Cell 107, 855-867<br>hIgG1-FcSpyCatcher3, catalog number: TZC009, Biorad
Peak calling parameters	Peaks were called against the negative control using Homer 4.9 with the command<br>findPeaks -style factor -F 6
Data quality	PCR duplicates were removed using Picard MarkDuplicates, and n/a<br>All peaks are at least 6-fold over input. There are 653 peaks for CLOCK:BMAL1 IP and 1434 for MYC:MAX IP. peaks were then searched for the E-box motif by MEME and only peaks with motif were retained.
Software	MEME-suite was used to find motifs within peaks and<br>R was used to plot read distribution at motif sites using the libraries VplotR 1.0.0. , rtracklayer 1.50. and ggplot2 3.3.6.<br><br>custom code can be accessed at:<br>https://github.com/nikolas848/eggers_2023_nature
